# Supplementary material for: Revealing the biophysics of lamina-associated domain formation by integrating theoretical modeling and high-resolution imaging
Source: Nat Commun. 2025 Aug 25;16:7909. doi: 10.1038/s41467-025-63244-1 (PMC12378204; doi:10.1038/s41467-025-63244-1)
Supplement: Supplementary file 1 — Supplementary Information [file 41467_2025_63244_MOESM1_ESM.pdf]

SUPPLEMENTARY INFORMATION

For

Revealing the Biophysics of Lamina-Associated Domain Formation by Integrating Theoretical Modeling and High-Resolution Imaging

Monika Dhankhar,[1,2,†] Zixian Guo,[1,3,†] Aayush Kant,[1,2,†] Ramin Basir,[1,2] Rohit Joshi,[1,2] Vinayak Vinayak,[1,2] Su Chin Heo,[4,5,6] Robert L. Mauck,[1,4,5,6] Melike Lakadamyali,[1,7] Vivek B. Shenoy [1,2,3,*]

[1] Center for Engineering Mechanobiology, University of Pennsylvania, Philadelphia, PA, 19104, USA-

[2] Department of Materials Science and Engineering, University of Pennsylvania, Philadelphia, PA, 19104, USA

[3] Department of Mechanical Engineering and Applied Mechanics, University of Pennsylvania, Philadelphia, PA, 19104, USA

[4] Department of Bioengineering, University of Pennsylvania, Philadelphia, PA, 19104, USA

[5] McKay Orthopaedic Research Laboratory, Department of Orthopaedic Surgery, Perelman School of Medicine, University of Pennsylvania, Philadelphia, PA, 19104, USA

[6] Translational Musculoskeletal Research Centre, Corporal Michael J, Crescenz VA Medical Centre, Philadelphia, PA 19104, USA

[7] Department of Physiology, Perelman School of Medicine, University of Pennsylvania, Philadelphia, PA 19104, USA

[†] Equal Contribution

[*] Corresponding author: vshenoy@upenn.edu

1 Details of Methods

1.1 Mathematical description of chromatin organization in the nucleus

To investigate the organization of chromatin in the nucleus, we develop a mathematical model for the phase separation of heterochromatin and euchromatin considering the chromatin-chromatin interactions, chromatin-lamina interactions, and epigenetic regulation of chromatin via histone acetylation or methylation. The nucleus is conceptualized as comprising three components: the nucleoplasm, heterochromatin, and euchromatin. At a given spatial coordinate $\boldsymbol{x}$ and time t the nuclear constitution is characterized by local volume fractions: $\phi_h(\boldsymbol{x},t)$ for heterochromatin, $\phi_e(\boldsymbol{x},t)$ for euchromatin and, $\phi_n(\boldsymbol{x},t)$ for nucleoplasm, with a stipulation that $\phi_e + \phi_h + \phi_n = 1$. This constraint ensures conservation of mass within the system. The physical state at any given location is thus discernible through two independent parameters: the volume fraction of nucleoplasm $\phi_n(\boldsymbol{x},t)$, and the net difference in volume fractions between heterochromatin and euchromatin, denoted as $\phi_d(\boldsymbol{x},t) = \phi_h(\boldsymbol{x},t) - \phi_e(\boldsymbol{x},t)$. The variable ϕ_d serves as an order parameter, signaling the dominance of either phase: negative values indicate a prevalence of euchromatin, while positive values denote a higher concentration of heterochromatin.

1.2 Free energy landscape of the nucleus

In terms of the independent variables $\phi_d(\boldsymbol{x},t)$ and $\phi_n(\boldsymbol{x},t)$, the free energy density at any point $\boldsymbol{x}$ in the nucleus can be expressed as $f(\phi_n, \phi_d, \nabla\phi_n, \nabla\phi_d)$. This formulation incorporates the energetic considerations associated with the interfaces between phases through the gradients of the volume fractions. Specific form of the free energy can be invoked by considering the various energetic contributions in the nucleus such as,

$$f = \underbrace{\frac{C}{2}\left[\phi_e^2 + \phi_h^2(\phi_{h0} - \phi_h)^2\right]}_{\text{chromatin-chromatin interactions}} + \underbrace{\frac{\kappa}{2}\left[|\nabla\phi_n|^2 + |\nabla\phi_d|^2\right]}_{\text{Interfacial energy}} - \underbrace{\frac{V(\phi_h)}{d_0}\phi_h e^{-\frac{d}{d_0}}}_{\text{chromatin-lamina interactions}} . \quad \text{(S1)}$$

The first term in Eq S1 emerges from the interplay between the entropy and the enthalpy of mixing for the heterochromatin and euchromatin phases, conferring a double-well configuration to the free energy landscape, as shown in the contour plot in Fig. 1E. The two wells, shown as blue and red dots, are the energy minima corresponding to the two stable phases of chromatin - a water-rich, loosely packed euchromatin phase ($\phi_h = 0$) or a compacted water-poor heterochromatin phase ($\phi_h = \phi_{h0}, \phi_n \sim 0$). Here ϕ_{h0} is maximum heterochromatin amount and denotes the extent of compaction in the heterochromatin phase. An initial chromatin state, indicated by a white dot, will naturally phase separate into domains of heterochromatin and euchromatin (white arrows).

The second term denotes the energy penalty associated with forming phase boundaries between the euchromatin and heterochromatin as they separate. The term κ is the increase in the energy due to formation of a unit width of the interface. As κ increases, there is a greater penalty on formation of sharp interfaces, resulting in more smooth interfaces which are wider. Thus κ directly control the width and the energy of the phase boundaries. Note that the term $|\nabla\phi_{n,d}|$ is the magnitude of the slope of the interface.

The last term captures the interactions between the chromatin and the lamina via chromatin anchoring proteins (HDAC3, LAP2β, emerin)[1, 2] with function $V(\phi_h)$ (refer Supplementary information 5.1 for details) denoting the strength of these anchoring interactions per unit area. These interactions are strongest at the nuclear periphery (distance from lamina $d = 0$) and vanish exponentially over a length scale d_0, comparable to size of anchoring proteins.

The total free energy of the nucleus can be written as,

$$\Pi[\phi_n, \phi_d] = \int_{\mathrm{V_n}} f(\phi_n, \phi_d, \nabla\phi_n, \nabla\phi_d) dV - \int_{\partial V_p} \bar{\mu}_n I^n dA \,. \tag{S2}$$

Here, $\mathrm{V_n}$ is the volume of nucleus and $\partial \mathrm{V}_p$ denotes the surface of the nucleus where water exchange between nucleus and cytoplasm takes place. $\bar{\mu}_n$ is external chemical potential and I^n is the volume of water entering per unit surface area into the nucleus.

The energy landscape described by Eq 3 (Eq S1) guides the chromatin's temporal dynamics from an initial configuration (white dot in Fig. 1E) into the two energy minima, corresponding to the two chromatin phases (red and blue dot). The spatiotemporal evolution of nucleoplasm and chromatin occurs such that the free energy of chromatin organization is driven towards the energy minima. The driving forces for this evolution are called the chemical potential (μ_n and μ_d) of nucleoplasm and epigenetic marks respectively and are quantitatively dependent on the gradients in the energy landscape. Using variational principles, these chemical potentials are written as,

$$\begin{aligned} \mu_n(\boldsymbol{x}, t) &= \frac{\delta f}{\delta \phi_n} = \frac{\partial f}{\partial \phi_n} - \boldsymbol{\nabla} \cdot \left(\frac{\partial f}{\partial \nabla \phi_n}\right) \\ \mu_d(\boldsymbol{x}, t) &= \frac{\delta f}{\delta \phi_d} = \frac{\partial f}{\partial \phi_d} - \boldsymbol{\nabla} \cdot \left(\frac{\partial f}{\partial \nabla \phi_d}\right) \end{aligned} \,. \tag{S3}$$

Here, μ_n is the chemical potentials of nucleoplasm driving its kinetics as described in Supplementary Information section 1.3 and μ_d is the chemical potential for the order parameter ϕ_d evolving the epigenetic marks in a conserved manner as discussed in the Supplementary Information section 1.3 and 1.4.

1.3 Diffusion and reaction kinetics

The dynamic evolution of the nucleus is governed by both diffusion and reaction kinetics. First, we consider the nucleoplasm, the inert content within the nucleus. Its conservative evolution at all points in the nucleus depends on the local flux of nucleoplasm. This flux, as governed by Fick's first law, is directly proportional to the gradient of its chemical potential as $\boldsymbol{\mathcal{J}}^n = -M_n \nabla \mu_n$, where M_n is the mobility of nucleoplasm in the nucleus. Thus, we can express the change in nucleoplasm volume fraction over time as a result of diffusion as

$$\frac{\partial \phi_n}{\partial t} = \underbrace{M_n \nabla^2 \mu_n}_{\text{diffusion}} \,. \tag{S4}$$

The physical interpretation of Eq S4 which regulates the local redistribution of water particles is discussed in Supplementary Information Section 1.4. On the other hand, the spatiotemporal evolution of chromatin – which distinguishes between the heterochromatin and the euchromatin phases – is governed by epigenetic reaction kinetics. The reactions facilitate a non-conservative regulation of the overall levels of histone methylation and acetylation. Heterochromatic phase, which is prominently methylated is converted into euchromatin phase via acetylation reaction involving the removal of methyl groups called demethylation followed by addition of acetyl group on the histone tails via acetyltransferase activity. The overall rate of acetylation reaction (at a rate Γ_{ac}) is a combination of its substeps such that $\frac{1}{\Gamma_{ac}} = \frac{1}{\Gamma_{HDM}} + \frac{1}{\Gamma_{HAT}}$ (Fig. 1D). Similarly, the euchromatin phase is converted into heterochromatin via an overall methylation reaction (at a rate Γ_{me}) incorporating its sub steps of histone deacetylation and methyltransferase activity such that

$\frac{1}{\Gamma_{me}} = \frac{1}{\Gamma_{HDAC}} + \frac{1}{\Gamma_{HMT}}$ (Fig. 1D). The reaction kinetics driven by the *epigen*etic regulation of overall acetylation and methylation result in the conversion of euchromatin phase into heterochromatin phase at a rate,

$$\left.\frac{\partial \phi_d}{\partial t}\right|_{\text{epigen}} = 2(\Gamma_{me}\phi_e - \Gamma_{ac}\phi_h)\,. \tag{S5}$$

However, the rates of reactions (Γ_{me} and Γ_{ac}) in Eq. S5 are not constant, but dependent on the strength of energetic interactions between neighboring nucleosomes, as we clarify next.

The chromatin-chromatin energy landscape has two stable wells as shown in Fig. 1E. The heterochromatic well (red in Fig. 1E) is marked by high ϕ_h (~ 0.7), which indicates that a high volume of heterochromatic nucleosomes, i.e. tightly compacted heterochromatin, is energetically favored. The other well (blue in Fig. 1E) is rich in water with euchromatic nucleosomes, with a minimal presence of heterochromatic nucleosomes. Thus, the energy landscape results in like-marked (heterochromatin-heterochromatin or euchromatin-euchromatin) neighbors being more stable than unlike-marked (heterochromatin-euchromatin) neighbors. When reactions convert heterochromatin into euchromatin, or vice-versa, the reactions that lead to the formation of more like-marked nucleosome neighbors will be preferred over the reactions that lead to more intermingling of heterochromatin and euchromatin. We have shown previously [3] and again schematically in Supplementary Information Section 1.4 below that such neighborhood dependent reaction kinetics effectively emulates a diffusion-like conservative evolution of epigenetic marks. In other words, the epigenetic marks are effectively spatially diffusing, resulting in coarsening of the heterochromatin and euchromatin phases (as shown schematically in Supplementary Fig. 1 and explained in Supplementary Information Section 1.4). This effectively *cons*ervative evolution can be written as,

$$\left.\frac{\partial \phi_d}{\partial t}\right|_{\text{cons}} = M_d \nabla^2 \mu_d\,. \tag{S6}$$

Here, M_d is the effective mobility of epigenetic marks in nucleus, the physical value of which we have previously estimated from experimentally observed mean-squared displacement curves [3]. The term μ_d in Eq. S6 denotes the effective chemical potential of epigenetic marks that drives the diffusion-like epigenetic evolution and is dependent on the free energy of chromatin organization, as described in Eq S3.

Combining these dynamics (Eq S5-S6), the overall evolution of the order parameter ϕ_d can be written as,

$$\frac{\partial \phi_d}{\partial t} = 2\,\underbrace{(\Gamma_{me}\phi_e - \Gamma_{ac}\phi_h)}_{\text{epigentic regulation}} + \underbrace{M_d \nabla^2 \mu_d}_{\text{diffusion}}\,. \tag{S7}$$

This comprehensive framework of equations (S3), (S4), and (S7) provides a complete understanding of spatio-temporal evolution of chromatin organization within the nucleus.

1.4 Physical interpretation of diffusion and reaction kinetics

As discussed in Supplementary Information Section 1.3, we have incorporated the diffusion kinetics of nucleoplasm as well as the reaction kinetics of epigenetic regulation in our model. Mathematically, these are described by Eqs S4 and S7. In this section we the physical interpretation of the kinetics underlying diffusion and reaction driven chromatin evolution.

The nucleoplasm kinetics, given by Eq S4 is diffusion driven and conservative, i.e. unless there is a flux of water into or out of the nucleus, the total amount of nucleoplasm in the nucleus remains constant. The favorable energetic interactions, such as those mediated by nucleosome bridging proteins such as HP1α [4], results in methylated histones coming together. This compaction of chromatin requires local movement of water molecules away from the condensing heterochromatin domains, as shown in Supplementary Fig. 1. However, this spatiotemporal evolution does not alter the total number of water molecules. This energetic driven local conservative movement of water molecules is captured via the diffusion of nucleoplasm in Eq S4.

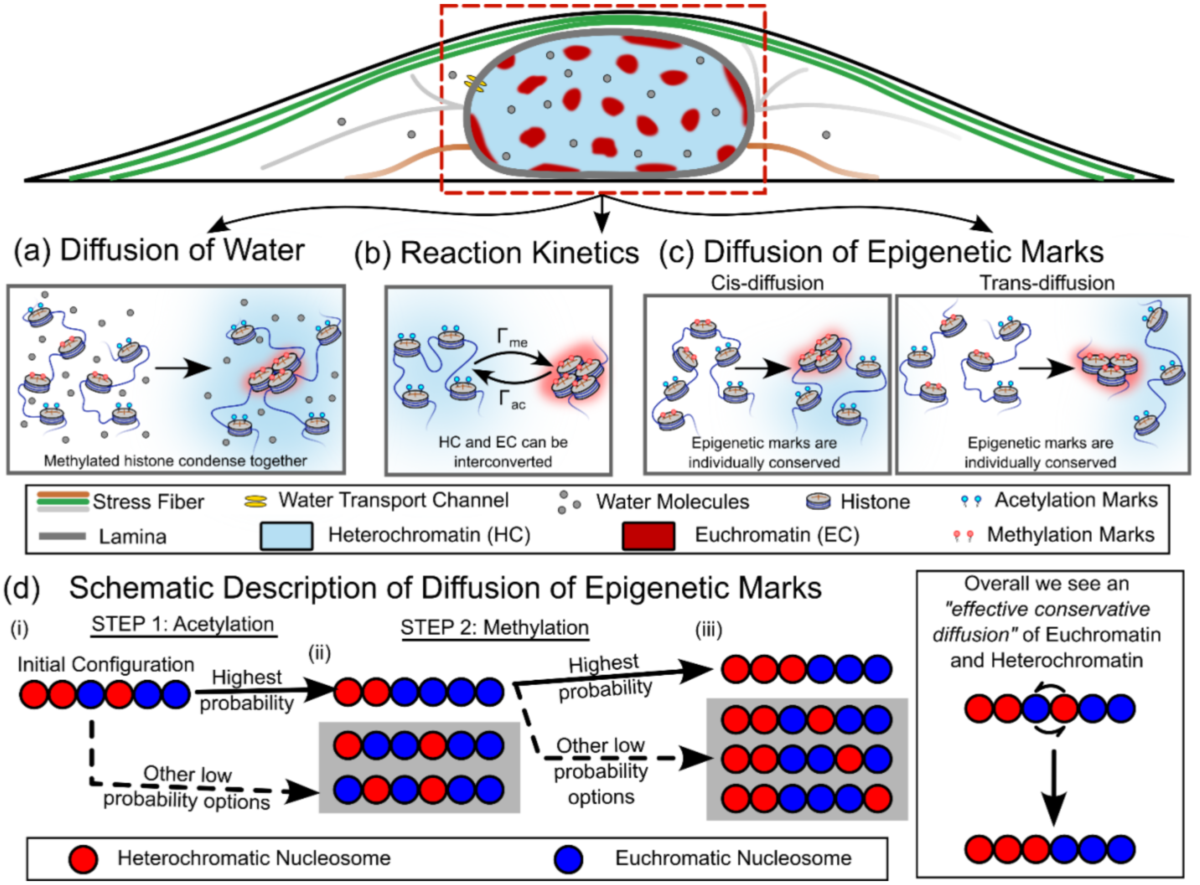

Supplementary Fig. 1: A schematic depicting the individual roles of the diffusion and reactions kinetics incorporated into the heterochromatin organization model. (a) Conservative diffusion of water which can redistribute molecules of water within the nucleus without changing the total amount of water, or total amount of hetero- or euchromatin in the nucleus. (b) Non-conservative reaction kinetics of histone acetylation and methylation, which allows an interconversion of chromatin phases. This changes the individual amounts of heterochromatin and euchromatin in the nucleus without changing the total amount of DNA. The reaction rates determine the ratio of heterochromatin to euchromatin at steady state. (c) The chromatin-chromatin interactions contribute to the reaction kinetics effectively driving a conservative evolution of epigenetic marks, which we call 'diffusion of epigenetic marks'. (d) The diffusion of epigenetic marks is an overall result of reaction kinetics coupled with preference of like-like neighbor over unlike ones, thereby effectively rendering reactions ate certain sites more probable than others. Adapted from Ref[3].

The kinetics of chromatin evolution (Eq S7) involves epigenetic regulation via the methylation or acetylation reactions and gives rise to the reaction kinetics (first term in Eq S7). The reaction kinetics are non-conservative as they involve interconversion of acetylated histones to methylated histones (methylation kinetics, Γ_{me}) and vice versa (acetylation kinetics, Γ_{ac}) as shown in Supplementary Fig. 1.

However, the reaction kinetics given by the first term in Eq S7 does not take into account the energetics of chromatin-chromatin interactions. As described in Eq S1.2, the chromatin energy landscape results in heterochromatin-heterochromatin or euchromatin-euchromatin neighbors being more stable than neighbors that are not like marked. As a result, if the methylation reaction results in conversion of a heterochromatin-heterochromatin neighbor into a heterochromatin-euchromatin neighbor, such conversion is energetically unfavorable. On the other hand, if reactions convert heterochromatin-euchromatin neighbors into a heterochromatin-heterochromatin or a euchromatin-euchromatin pair it is energetically favorable. Thus, the effective rates of epigenetic reactions are determined by the specific locations of the nucleosome where reactions occur dependent on the epigenetic marks of the neighboring nucleosomes.

A schematic evolution of epigenetic marks driven by such neighborhood dependent reaction rates is shown in Supplementary Fig. 1. At a given time, a portion of chromatin polymer has epigenetic marks as shown in Supplementary Fig.1 (red: heterochromatin, blue: euchromatin). Let the first step involve acetylation reaction – converting a heterochromatic nucleosome into euchromatic form. While such conversion could happen at any of the nucleosomes, energetic interactions favor like-marked neighbors such that the configuration shown in Supplementary Fig. 1. has the highest probability of occurrence. If the next step involves further acetylation, in our model it will be captured by the non-conservative first term in Eq S7. So, we only consider the case if the next step involves methylation – such that the number of heterochromatic and euchromatic nucleosomes are individually conserved. Again, due to energetic interactions favoring like-marked nucleosomes the highest probability of occurrence is for the configuration as shown in Supplementary Fig.1. Thus, over the course of two steps in chromatin kinetics, we see that neighborhood dependent reaction kinetics can effectively result in a 'conservative' spatiotemporal evolution which leads to the coarsening of the two phases of chromatin. Such conservative evolution is effectively captured in our coarse-grained model via the second term in Eq S7 called the 'diffusion of epigenetic marks'.

Note that during the sequence of events described here, and depicted in Supplementary Fig. .1d, the epigenetic marks (i.e. red and blue beads in Supplementary Fig. 1d) are effectively diffusing along the chain of the chromatin polymer. This is an effective cis-diffusion and is also depicted in the schematic cartoon shown in Supplementary Fig. 1c, where the red-marked heterochromatic nucleosomes moved along the polymer to compact together to form the heterochromatic domain. However, in three-dimensions this effective diffusion need not be limited to along the polymer chain. For example, consider three red-marked heterochromatic nucleosomes which are not neighbors along the polymer chain, but are spatially close due to the three-dimensional arrangement of the chromatin polymer (as depicted in schematic cartoon in Supplementary Fig. 1c). Analogous to the 1D example in Supplementary Fig. 1d, the epigenetic reactions may (in a two-step process) convert heterochromatin and euchromatin such that the epigenetic marks effectively diffuse across the chain, which we called a trans-diffusion of epigenetic marks. The diffusion term in our coarse-grained model (Eq S7, second term) is isotropic and involves both cis- and trans- diffusion mechanisms. The quantitative equivalence of the diffusion term used in Eq S7 and the neighborhood dependent reaction kinetics has been previously shown by us [3].

1.5 Rescaling the equations for numerical implementation

The governing equations derived in Supplementary Information Section 1.2 and 1.3 show an intrinsic length and time scale, which can be used for rescaling the equations. The rescaled equations are used in the numerical implementation of the solution of the governing equations. Here, we demonstrate the steps involved in obtaining a rescaled, non-dimensional set of governing equations by leveraging these inherent scales.

The reaction-diffusion kinetics described in Eq S7 yield a characteristic length scale determined by interplay of diffusion and reaction rates i.e. $\ell_{RD} = \sqrt{M_d/\Gamma_{ac}} = \sqrt{D/\Gamma_{ac}}$. Remarkably, another intrinsic length scale arises from the competition between interfacial and bulk mixing energies from Eq S1, i.e. the width of the interface $\ell_{int} = \sqrt{\kappa/c}$. In our simulations and theoretical analyses (discussed in Supplementary Information Section 3), we find that the reaction-diffusion length significantly influences the sizes and spacing of heterochromatin domains. Consequently, we opt to rescale all lengths relative to ℓ_{RD}, such that $\tilde{x} = x/\ell_{RD}$. Furthermore, the reaction rates provide an inherent time scale for the system. Therefore, we rescale all times as $\tilde{t} = t\Gamma_{ac}$. The coefficient c , representing the energy of chromatin-chromatin interactions in Eq (S1), serves as an energy scaling factor. Thus, all energy densities are rescaled as $\tilde{f} = f/c$.

Rescaling Eq S1, we obtain

$$\tilde{f} = \underbrace{\frac{1}{2}\left[\phi_e^2 + \phi_h^2(\phi_\mathrm{h}^\mathrm{max} - \phi_h)^2\right]}_{\text{chromatin-chromatin interactions}} - \underbrace{\frac{\tilde{V}(\phi_h)}{d_0}\phi_h e^{-\frac{d}{d_0}}}_{\text{chromatin-lamina interactions}} + \underbrace{\frac{\delta^2}{2}|\nabla\phi_n|^2 + \frac{\delta^2}{2}|\nabla\phi_d|^2}_{\text{Interfacial energy}} . \tag{S8}$$

Here, $\tilde{V}(\phi_h) = V(\phi_h)/c$ is the rescaled strength of chromatin-lamina anchoring interactions. The parameter $\delta = \frac{\ell_{int}}{\ell_{RD}}$ is a rescaled measure of the width of the interface.

The rescaled chemical potentials from Eq S3 expand as,

$$\tilde{\mu}_n(\tilde{\boldsymbol{x}}, \tilde{t}) = -\phi_e - \phi_h(\phi_{h0} - \phi_h)(\phi_{h0} - 2\phi_h) - \frac{1}{2}\frac{\tilde{V}(\phi_h)}{d_0}e^{-\frac{d}{d_0}} - \delta^2\nabla^2\phi_n ,$$
$$\tilde{\mu}_d(\tilde{\boldsymbol{x}}, \tilde{t}) = -\phi_e + \phi_h(\phi_{h0} - \phi_h)(\phi_{h0} - 2\phi_h) - \frac{1}{2}\frac{\tilde{V}(\phi_h)}{d_0}e^{-\frac{d}{d_0}} - \delta^2\nabla^2\phi_d . \tag{S9}$$

Lastly, we rescale the kinetics equations such that,

$$\frac{\partial\phi_n}{\partial\tilde{t}} = \underbrace{\nabla^2\tilde{\mu}_n}_{\text{diffusion}} ,$$
$$\frac{\partial\phi_d}{\partial\tilde{t}} = \underbrace{\nabla^2\tilde{\mu}_d}_{\text{diffusion}} + \underbrace{2(\tilde{\Gamma}_{me}\phi_e - \phi_h)}_{\text{epigenetic regulation}} . \tag{S10}$$

Here, all reaction rates have been rescaled with respect to the time scale such that $\tilde{\Gamma}_{me} = \frac{\Gamma_{me}}{\Gamma_{ac}}$. These rescaled equations, Eq (S8) and Eq (S10), are simultaneously solved numerically using COMSOL Multiphysics, subject to boundary conditions ensuring no flux of nucleoplasm or epigenetic marks across all boundaries (see Supplementary Information section 8 for more details).

2 Average chromatin phase contents is determined by epigenetic reactions

Here we show that the average amount of eu- and heterochromatin in nucleus is determined solely by the epigenetic reactions. The spatiotemporal evolution of order parameter ϕ_d due to diffusion of epigenetic marks and kinetics of interconversion of eu- and heterochromatin phases, as described by Eq S7 as,

$$\frac{\partial \phi_d}{\partial t} = M_d \nabla^2 \mu_d + 2(\Gamma_{me}\phi_e - \Gamma_{ac}\phi_h)\,. \tag{S11}$$

At steady state, $\frac{\partial \phi_d}{\partial t} = 0$, the relation between the average euchromatin content $\bar{\phi}_e$ and the average heterochromatin $\bar{\phi}_h$ content in the nucleus can be obtained by averaging Eq S11 as,

$$0 = M_d \frac{\int_{V_n} \nabla^2 \mu_d \, dV}{\int_{V_n} dV} + 2(\Gamma_{me}\bar{\phi}_e - \Gamma_{ac}\bar{\phi}_h)\,.$$

The term $\nabla^2 \mu_d$ (first term on the right-hand side) on averaging over the entire volume of the nucleus can be approximated to zero. Thus,

$$0 = 2(\Gamma_{me}\bar{\phi}_e - \Gamma_{ac}\bar{\phi}_h)\,.$$

Given that $\phi_e + \phi_h + \phi_n = 1$, we obtain the following relationship:

$$(\Gamma_{me} + \Gamma_{ac})\bar{\phi}_h = \Gamma_{me}(1 - \bar{\phi}_n)\,,$$

Or,

$$\bar{\phi}_h = \frac{\Gamma_{me}\,(1 - \bar{\phi}_n)}{\Gamma_{me} + \Gamma_{ac}}, \qquad \bar{\phi}_e = \frac{\Gamma_{ac}\,(1 - \bar{\phi}_n)}{\Gamma_{me} + \Gamma_{ac}}\,. \tag{S12}$$

After rescaling, the average eu- and heterochromatin contents obtained via Eq S12 can be expressed as

$$\bar{\phi}_h = \frac{\tilde{\Gamma}_{me}\,(1 - \bar{\phi}_n)}{\tilde{\Gamma}_{me} + 1}, \qquad \bar{\phi}_e = \frac{(1 - \bar{\phi}_n)}{\tilde{\Gamma}_{me} + 1}\,. \tag{S13}$$

3 Characteristic Heterochromatin Domain size determination in presence of Reactions

The reaction kinetic parameters $(\Gamma_{me}, \Gamma_{ac})$ not only determine the average amounts of heterochromatin and euchromatin in the nucleus (Eq S12, S13), but also influence the sizes of the heterochromatin domains, as shown in Section 2.2 of the main manuscript. Here, we show the detailed derivation of interior heterochromatin domain size away from nuclear periphery. To analyze the steady state size of the heterochromatin domain, we first examine the chromatin composition within and around the droplet (Supplementary Fig. 2). As discussed in Supplementary Information Section 2, the acetylation and methylation together determine the mean heterochromatin (and euchromatin) content in the nucleus, given by Eq S12. In absence of any energetic considerations, this would give rise to a homogeneous mean chromatin composition $(\bar{\phi}_h, \bar{\phi}_e)$. However, this composition (Fig. 1E; white circle) lies in neither of the energy wells and is thus energetically unfavorable. To reduce the total free energy, the system phase separates via nucleation of heterochromatin droplets (Supplementary Fig. 2).

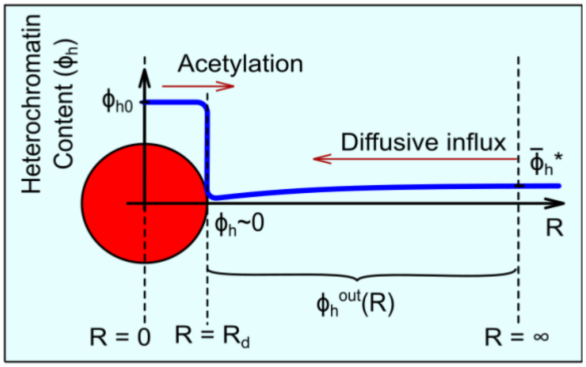

Supplementary Fig. 2: The competition of diffusion driven influx of heterochromatin with the epigenetic reaction driven outflux of heterochromatin from the heterochromatin domain determines its steady state size. The Figure also shows the radial distribution of heterochromatin volume fraction ϕ_h in and around the domain.

Due to phase separation, the heterochromatin volume fraction immediately outside the droplet is $\phi_h \approx 0$ corresponding to the euchromatic energy well. Far away from the droplet, the mean composition $(\bar{\phi}_h, \bar{\phi}_e)$ remains undisturbed. However, when there is a significant peripheral sequestering of methylated histones, the far-field concentration of heterochromatin $\bar{\phi}_h^*$ (Supplementary Fig. 2), drops from the average concentration $\bar{\phi}_h$ (discussed in Supplementary Information Section 4).

In the dilute limit, where there is significantly more euchromatin than heterochromatin, the droplet size can be considered much smaller than the spacing between domains such that neighboring droplets are far enough to not interact with each other. Under such assumption the heterochromatin distribution becomes spherically symmetric. By adopting a polar coordinate system with origin at the droplet center, we can write the steady-state concentration field $\phi_h(R)$ as a function of distance to the center of the droplet, R as

$$\phi_h(R) = \begin{cases} \phi_{h0} & R < R_d \\ \phi_h^+ = \dfrac{\kappa}{R_d} & R = R_d^+ \\ \bar{\phi}_h^* & R = \infty \end{cases} ,$$

where R_d is the radius of the droplet and κ is the measure of interfacial energy. Supplementary Fig. 2 shows the distribution of heterochromatin content $\phi_h(R)$ around a spherical heterochromatin domain (red) of radius R_d as it grows surrounded by euchromatin phase (in light blue). At steady state, heterochromatin composition outside the droplet follows the equation,

$$D_h \nabla^2 \phi_h^{out} - \Gamma_{ac}\phi_h^{out} + \Gamma_{me}\phi_e = 0 \,. \qquad \text{(S14)}$$

Here $D_h = M_h c$ is the diffusivity of heterochromatin in the nucleoplasm. By solving Eq S14 outside the heterochromatin domain, with boundary conditions $\phi_h|_{R_d^+} = \kappa/R_d$ and $\phi_h|_\infty = \bar{\phi}_h^*$, we get

$$\phi_h^{out}(R) = \bar{\phi}_h^* + (\phi_h^+ - \bar{\phi}_h^*)\frac{R_d}{R} e^{\frac{R_d - R}{\ell_{RD}}} ,$$

where ℓ_{RD} is the characteristic reaction-diffusion length scale given under a dilute limit as $\ell_{RD} = \sqrt{\frac{D_h}{\Gamma_{ac}}}$. Thus,

$$\frac{\partial \phi_h^{out}}{\partial R} = (\bar{\phi}_h^* - \phi_h^+)\frac{R_d(\ell_{RD} + R)}{\ell_{RD} R^2} e^{\frac{R_d - R}{\ell_{RD}}} .$$

Thus,

$$\left.\frac{\partial \phi_h^{out}}{\partial R}\right|_{R \to R_d} = \frac{\bar{\phi}_h^*}{R_d} - \frac{\kappa}{R_d^2} \qquad \left(when \frac{R_d}{\ell_{RD}} \ll 1\right). \qquad \text{(S15)}$$

Having obtained the heterochromatin concentration field, we next discuss the growth dynamics of heterochromatin droplet. The droplet grows due to the reaction-diffusion driven influx (blue curve in Supplementary Fig. 2) of heterochromatin. On the other hand, within the heterochromatin droplet (with $\phi_h = \phi_{h0}$) histone acetylation reactions will allow conversion of heterochromatin inside the droplet into euchromatin outside. This acetylation driven outflux oppose the diffusive influx of heterochromatin and thereby reduce the size of the droplet (Supplementary Fig. 2). Thus, the rate of change of the volume of the droplet V_d can be written as,

$$\frac{dV_d}{dt} = \frac{4}{3}\pi\frac{dR_d^3}{dt} = \underbrace{J^{in}}_{\text{inwards diffusion}} - \underbrace{\Gamma_{ac} \times \frac{4}{3}\pi R_d^3 \phi_{h0}}_{\text{Acetylation}} , \tag{S16}$$

where the diffusive influx of heterochromatin is,

$$J^{in} = 4\pi R_d^2 D_h \frac{\partial \phi_h^{out}}{\partial R}\bigg|_{R\to R_d} . \tag{S17}$$

Using the spatial gradient from Eq S15, we simplify Eq S16 as,

$$4\pi R_d^2 \frac{dR_d}{dt} = 4\pi D_h(\bar{\phi}_h^* R_d - \kappa) - \Gamma_{ac} \times \frac{4}{3}\pi R_d^3 \phi_{h0} ,$$

$$\frac{dR_d}{dt} = D_h\left(\frac{\bar{\phi}_h^*}{R_d} - \frac{\kappa}{R_d^2}\right) - \frac{\Gamma_{ac}}{3} R_d \phi_{h0} . \tag{S18}$$

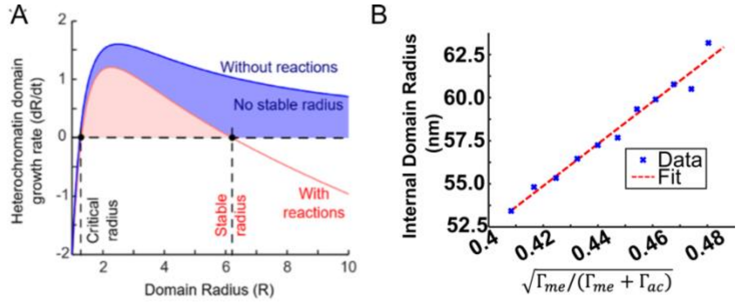

Supplementary Fig. 3: (A)The growth rate of the heterochromatin domain. (B) The scaling relationship between the size of interior HC domains and the level of methylation.

Using Eq S18, we plot the rate of change of heterochromatin domain size with respect to the instantaneous domain radius (Supplementary Fig. 3A). Above a critical radius, all heterochromatin domains grow ($dR_d/dt > 0$). In the absence of reactions ($\Gamma_{ac} = 0$, blue curve), the rate of change in the heterochromatin domain radius is always positive indicating that the domain will keep growing as long as its radius is larger than the critical radius. However, in the presence of the reactions, the domains grow until their growth rate reaches a zero value. This gives the stable size of heterochromatin domains. The domains larger than the stable radius will shrink back to the stable radius. The stable radius (R_d^s) can be obtained by setting $dR_d/dt = 0$ in Eq S18 such that,

$$0 = \frac{D_h \bar{\phi}_h^*}{\phi_{h0}} - \frac{\Gamma_{ac}}{3} {R_d^s}^2 .$$

Thus,

$$R_d^s = \sqrt{\frac{3D_h}{\Gamma_{ac}\phi_{h0}}\bar{\phi}_h^*}. \tag{S19}$$

On rescaling, Eq S19 becomes,

$$\tilde{R}_d^s = \sqrt{\frac{3}{\phi_{h0}}\bar{\phi}_h^*}. \tag{S20}$$

All lengths are rescaled with respect to ℓ_{RD}, while all times with respect to $1/\Gamma_{ac}$. Eq S20 give a non-dimensional dependence of heterochromatin domain size on the epigenetic kinetics.

In deriving the domain size scaling (Eq S19), we have considered a 3-dimensional scenario where spheroids of heterochromatin domains are formed. However, for consistency with our simulations and the STORM images, the derivation can be easily repeated for the 2D case – to obtain the same scaling relations as in 3D case. In 2D, Eq. S14 can be rewritten as

$$D_h\left(\frac{\partial^2\phi_h}{\partial r^2}+\frac{1}{r}\frac{\partial\phi_h}{\partial r}\right)-\Gamma_{ac}\phi_h+\Gamma_{me}\phi_e=0\,,$$

which is the modified Bessel's equation of order 0. The solution to this equation is

$$\phi_h=\bar{\phi}_h^*+(\phi_h^+-\phi_{h0})\frac{K_0(r/l_{RD})}{K_0(R_d/l_{RD})},$$

where K_0 denotes the zeroth order Bessel K function. Also, in 2D Eq. S16 becomes

$$\frac{dV_d}{dt}=2\pi R_d\frac{dR_d}{dt}=\underbrace{-2\pi R_d D_h\frac{\partial\phi_h^{out}}{\partial r}}_{diffusive\ flux}-\underbrace{\pi R_d^2\Gamma_{ac}}_{acetylation}\phi_{h0}. \quad \text{(S21)}$$

Solving Eq. S21 at the steady state $\frac{dV_d}{dt}=0$ yields a scaling relation between the radius of heterochromatin domain and the level of methylation,

$$\frac{K_1(R_d^s/l_{RD})}{K_0(R_d^s/l_{RD})}\sim\frac{2D_h}{\Gamma_{ac}\phi_{h0}}\,\bar{\phi}_h^*\,.$$

Thus, we obtain the same scaling relation $R_d^s=f\left(\frac{D_h}{\Gamma_{ac}\phi_{h0}}\bar{\phi}_h^*\right)$, where f is a function determined from Eq. S21. In the range of our parameters, $R_d^s\sim\sqrt{\frac{D_h}{\Gamma_{ac}\phi_{ho}}\bar{\phi}_h^*}$ provides a good approximation for the scaling of the size of heterochromatin domains, as confirmed by our numerical simulations (Supplementary Fig. 3B).

As discussed in the beginning of this section, $\bar{\phi}_h^*$ is the far-field heterochromatin concentration for the interior of the nucleus. The peripheral sequestering of heterochromatin towards the nuclear lamina governs this value, as we discuss next.

4 Far-field Heterochromatin concentration is influenced by Peripheral HC Sequestering

The competition between interior and peripheral heterochromatin domains for the available DNA pool within the nucleus shapes the overall heterochromatin distribution. As discussed in Section 2.2 of the main manuscript, this competition is expressed as:

$$Total\ HC=HC^{interior}+HC^{Periphery}. \quad \text{(S22)}$$

Therefore, when there is a significant peripheral sequestering of heterochromatin, the far-field concentration of heterochromatin $\bar{\phi}_h^*$ (Supplementary Fig. 2), drops from the reaction rate determined average concentration $\bar{\phi}_h\approx\frac{\Gamma_{me}(1-\bar{\phi}_n)}{\Gamma_{me}+\Gamma_{ac}}$.

In Eq S22, 'Total HC' is the average heterochromatin content in the nucleus $\bar{\phi}_h$ times the total nuclear area, $HC^{interior}$ is obtained by total number of interior domain times the area of one domain and $HC^{Periphery}$ is an estimate of heterochromatin accumulated at periphery, such that

$$\underbrace{\pi R_{nuc}^2}_{\substack{Area\ of\\ Nucleus}} * \underbrace{\bar{\phi}_h}_{\substack{Average\ HC\\ in\\ Nucleus}} = \underbrace{\frac{\alpha\pi R_{nuc}^2}{\pi l_{RD}^2}}_{\substack{Number\ of\\ interior\ HC\\ domains}} * \underbrace{\pi R_d^2}_{\substack{Area\ of\ one\\ interior\ HC\\ domain}} + \underbrace{2\pi R_{nuc}}_{\substack{Periphery\ of\\ Nucleus}} * \underbrace{T_{LAD}}_{\substack{Thickness\ of\\ peripheral\ HC}} . \tag{S23}$$

Here, R_{nuc} is the radius of the nucleus and T_{LAD} is the size of peripheral heterochromatin domain (details in Supplementary Information Section 5). The parameter α, analogous to the atomic packing factor, gives an estimate of fraction of volume occupied by the heterochromatin in nucleus interior. On using Eq 19, Eq S23 gives

$$\bar{\phi}_h = \frac{3\alpha}{\phi_{ho}} \bar{\phi}_h^* + \frac{2T_{LAD}}{R_{nuc}},$$

Here $\frac{3\alpha}{\phi_{ho}} \approx 1$, thus

$$\bar{\phi}_h^* = \bar{\phi}_h - \frac{2T_{LAD}}{R_{nuc}}. \tag{S24}$$

On rescaling, Eq S24 becomes,

$$\bar{\phi}_h^* = \frac{\tilde{\Gamma}_{me}}{\tilde{\Gamma}_{me} + 1} - \frac{2\tilde{T}_{LAD}}{\tilde{R}_{nuc}}. \tag{S25}$$

Here, $\tilde{T}_{LAD}$ is the rescaled size of peripheral heterochromatin domain and $\tilde{R}_{nuc}$ is the normalized radius of the nucleus. Note that when the LAD thickness is very small $(\tilde{T}_{LAD} \ll \tilde{R}_{nuc})$, the influence of peripheral domains on interior domains is insignificant and $\bar{\phi}_h^* = \bar{\phi}_h$. The additional term $\frac{2\tilde{T}_{LAD}}{\tilde{R}_{nuc}}$ in Eq S25 arises from interdependence of interior and peripheral domains via Eq S22.

5 LAD morphology is regulated by chromatin-lamina affinity and epigenetic reactions

5.1 LAD shapes are determined by chromatin-lamina affinity

As discussed in section 4.2, the presence of chromatin-anchoring proteins such as LAP2β, HDAC3 and LBR [1, 5, 6] that sequester chromatin to the lamina is captured in our model through the chromatin-lamina interaction energy per unit surface area of nuclear lamina, $V(\phi_h)$ (Eq 3). These interactions lead to the localization of chromatin to the periphery, resulting in the formation of a euchromatin-heterochromatin (he) interface, a lamina-heterochromatin (lh) interface and a lamina-euchromatin (le) interface, as shown in Supplementary Fig. 4 (left panel). The surface tension γ of each of these interfaces is defined as the energetic cost of forming a unit area of the interface. The balance of the three surface tensions governs the extent of peripheral localization of chromatin, analogous to the wall-wetting phenomenon described by the well-known Young's equation, $\gamma_{lh} = \gamma_{he} cos\theta + \gamma_{le}$, where θ is the contact angle of the LAD with respect to the nuclear lamina, which determines the morphology of the LAD. Here, γ_{lh} and γ_{le} represents the surface tension at lamina-heterochromatin and lamina-euchromatin interface respectively, γ_{he} denotes the surface tension corresponding to heterochromatin-euchromatin interface (Supplementary Fig. 4). Thus, the contact angle of LAD with respect to the nuclear lamina θ, and consequently the LAD morphology, is determined by the energetic competition between chromatin-chromatin interactions and chromatin-lamina interactions as,

$$\theta = \cos^{-1}\left(\frac{\gamma_{lh} - \gamma_{le}}{\gamma_{he}}\right) = \cos^{-1}(V_{LAD}/\gamma_{he}).$$

Here, V_{LAD}/γ_{he} is the strength of chromatin-lamina interactions relative to the strength of chromatin-chromatin interactions, where $V_{LAD}(= V_{HC} - V_{EC} \sim \gamma_{lh} - \gamma_{le})$ quantifies the preferential interaction of lamina with heterochromatin over. Next, we drive the relation between surface tension (γ_{lh}) at lamina-chromatin interface and lamina-chromatin interaction strength(V_{LAD}).

The total free energy over the entire volume from Eq S1 can be written as

$$\Pi = \int_V \left[\Delta F(\phi_h, \phi_e) + \kappa\left[\left(\frac{d\phi_h}{dx}\right)^2 + \left(\frac{d\phi_e}{dx}\right)^2\right] + \underbrace{V(\phi_h^s)}_{energy/area}\ \delta(0)\right] dV. \tag{S26}$$

Here ϕ_h^s is the volume fraction of heterochromatin at the lamina interface. $V(\phi_h^s)$ is a general function, a measure of the chromatin-lamina interaction energy per unit area, with value V_{EC} when in euchromatin phase (when $\phi_h^s = 0$) and V_{HC} when in heterochromatin phase (when $\phi_h^s = 1$). We consider two regions – (i) where heterochromatin interacts with lamina, and (ii) where euchromatin interacts with lamina.

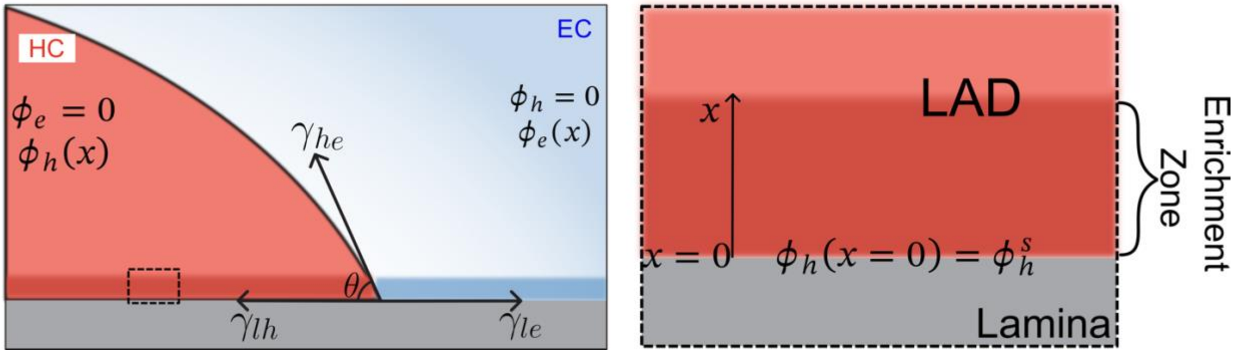

Supplementary Fig. 4: The balance of surface tensions at the interface of chromatin-lamina ($\gamma_{lh}$and γ_{le}) and the two phases of chromatin (γ_{he}) gives rise to a stable morphology of LADs, which have a characteristic contact angle with the lamina(θ). The right panel shows the zoomed in view of heterochromatin enriched zone at heterochromatin-lamina interface.

First consider the region (i) where $\phi_e = 0$. The total free energy in this region from Eq S26 is

$$\Pi = \int_0^x \left[\Delta F(\phi_h) + \kappa\left[\left(\frac{d\phi_h}{dx}\right)^2\right] + V(\phi_h^s)\delta(0)\right] dx. \tag{S27}$$

On simplification,

$$\Pi = V(\phi_h^s) + \int_0^x \left[\Delta F(\phi_h) + \kappa\left[\left(\frac{d\phi_h}{dx}\right)^2\right]\right] dx.$$

In region (i), the minima of total free energy Π occurs when,

$$\Delta F(\phi_h) = \kappa\left(\frac{d\phi_h}{dx}\right)^2. \tag{S28}$$

Thus, everywhere in the region (i):

$$\frac{d\phi_h}{dx} = \pm\sqrt{\frac{\Delta F(\phi_h)}{\kappa}}. \tag{S29}$$

With boundary conditions (at x=0),

$$\frac{dV(\phi_h^s)}{d\phi_h^s} = 2\kappa \left.\frac{d\phi_h}{dx}\right|_{x=0} = \pm 2\sqrt{\kappa \Delta F(\phi_h^s)}.$$

Now the surface tension i.e the free energy at lamina-heterochromatin interface

$$\gamma_{lh} = \underbrace{V(\phi_h^s)}_{=V^{HC}} + \int_0^\infty \left[\Delta F(\phi_h) + \kappa\left[\left(\frac{d\phi_h}{dx}\right)^2\right]\right] dx.$$

Use Eq S28 and S29:

$$\gamma_{lh} = V^{HC} + \int_1^{\phi_h^s} 2\Delta F(\phi_h)\sqrt{\frac{\kappa}{\Delta F(\phi_h)}}\, d\phi_h\,,$$

$$\gamma_{lh} = \underbrace{V^{HC}}_{lamina\ interaction\ strength} + \underbrace{2\int_1^{\phi_h^s} \sqrt{\kappa \Delta F(\phi_h)}\, d\phi_h}_{Excess\ energy\ due\ to\ enrichment}. \tag{S30}$$

Similarly in region (ii),

$$\gamma_{le} = \underbrace{V^{EC}}_{lamina\ interaction\ strength} + \underbrace{2\int_1^{\phi_e^s} \sqrt{\kappa \Delta F(\phi_e)}\, d\phi_e}_{Excess\ energy\ due\ to\ enrichment}. \tag{S31}$$

Balancing the surface tensions:

$$\gamma_{he}\cos\theta = \gamma_{lh} - \gamma_{le},$$

$$\gamma_{he}\cos\theta = V_{HC} - V_{EC} = V_{LAD}.$$

Notably the integral in right side in Eq S30 and S31 accounting for the excess energy due to enrichment at interface is negligible. We are extracting this difference V_{LAD} – the preferential interactions of lamina with the heterochromatin over euchromatin.

5.2 *LADs thickness is regulated by both chromatin-lamina affinity and epigenetic reactions*

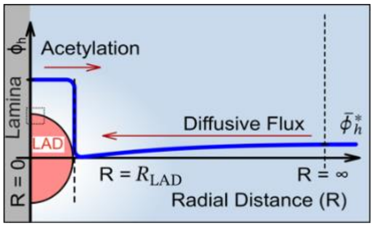

Supplementary Fig. 5: The formation of LADs in steady state requires a balance between diffusion-driven heterochromatin influx (into the LAD) and acetylation-driven conversion of heterochromatin into euchromatin.

As discussed in Section 2.2, chromatin-lamina affinity and epigenetic reactions synergistically determine the characteristic length-scales of the peripheral heterochromatin domains.

As in the interior of the nucleus, the free energy of chromatin organization can be lowered by nucleation of compacted heterochromatin domains ($\phi_h = \phi_{h0}$) at the nuclear periphery, immediately surrounded by euchromatin ($\phi_h = 0$). We consider the nucleation and growth of a single peripheral heterochromatin domain (Supplementary Fig. 5) of radius, R_{LAD}, at nuclear

periphery forming a contact angle θ with lamina. Far from the peripheral heterochromatin domain, the average heterochromatin volume fraction remains undisturbed at the reaction determined level $\bar{\phi}_h^*$. Similar to interior domains, at steady state, heterochromatin composition outside the peripheral domain follows the equation

$$D_h\left(\frac{\partial^2\phi_h}{\partial r^2}+\frac{1}{r}\frac{\partial\phi_h}{\partial r}\right)-(\Gamma_{ac}+\Gamma_{me})\phi_h+\Gamma_{me}=0, \tag{S32}$$

with solution

$$\phi_h=\bar{\phi}_h^*+(\phi_h^+-\phi_{h0})\frac{K_0(r/l_{RD})}{K_0\left(\frac{R_{LAD}}{l_{RD}}\right)}\approx\bar{\phi}_h^*\left[1-f\left(\frac{r}{l_{RD}},\frac{R_{LAD}}{l_{RD}}\right)\right].$$

Thus,

$$\frac{\partial\phi_h}{\partial r}|_{R_{LAD}}=\bar{\phi}_h^*\frac{\partial f}{\partial r}|_{R_{LAD}}.$$

Similar to interior domains, the concentration gradient driven diffusive influx (blue curve in Supplementary Fig. 5) is opposed by acetylation-driven effective outflux of methyl marks from within the peripheral domains. Thus, the rate of change of the LAD size A_{LAD} can be written as

$$\frac{dA_{LAD}}{dt}=\underbrace{D_h\int_0^{\theta}\frac{\partial\phi_h}{\partial r}|_{R_{LAD}}\,d\theta}_{influx}-\underbrace{A_{LAD}\Gamma_{ac}}_{outflux}. \tag{S33}$$

Note that, the reaction-diffusion influx depends on the heterochromatin-lamina contact angle θ and the peripheral heterochromatin domain size R_{LAD}/l_{RD}. Thus, at steady state

$$\frac{dA_{LAD}}{dt}|_{ss}=0=D_h\int_0^{\theta}\bar{\phi}_h^*\frac{\partial f}{\partial r}|_{R_{LAD}}\,d\theta-A_{LAD}\Gamma_{ac}.$$

On simplification,

$$0=D_h\bar{\phi}_h^*F\left(\frac{R_{LAD}}{l_{RD}},\theta\right)-R_{LAD}^2(\theta-sin\theta cos\theta)\Gamma_{ac}.$$

Thus

$$R_{LAD}=\sqrt{\frac{D_h}{\Gamma_{ac}}\bar{\phi}_h^*\frac{F\left(\frac{R_{LAD}}{l_{RD}},\theta\right)}{a(\theta)}}. \tag{S34}$$

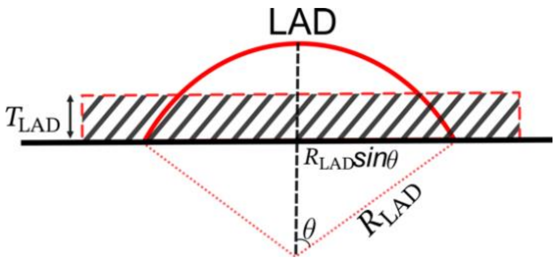

Supplementary Fig. 6: A schematic illustrating the transformation of LAD radius R_{LAD} into LAD thickness T_{LAD}.

Here, the function $a(\theta) = (\theta - sin\theta cos\theta)$ quantifies the area of the stable LAD. For a unified quantification of the individual peripheral domains, we define the length-scale of a LAD T_{LAD}, as its height averaged over its span as shown in Supplementary Fig. 6. Note that T_{LAD} is equivalent to the average thickness of the LAD and is inclusive of the previously defined LAD morphometric parameters θ and R_{LAD} such that

$$T_{LAD} = \frac{R_{LAD}^2 a(\theta)}{l_{RD}}. \qquad \text{(S35)}$$

So

$$T_{LAD} = \sqrt{\frac{D_h}{\Gamma_{ac}} \bar{\phi}_h^* F\left(\frac{R_{LAD}}{l_{RD}}, \theta\right)}.$$

The unified quantification of LADs via its length-scale T_{LAD} is particularly advantageous for quantifying the LADs observed via STORM images, where due to resolution limits, there may be a significant level of stochasticity in the measurement of individual contact angles of each LAD. Using $\bar{\phi}_h^*$ from Eq S24

$$T_{LAD} = \sqrt{\frac{D_h}{\Gamma_{ac}} F\left(\frac{R_{LAD}}{l_{RD}}, \theta\right)\left(\frac{\Gamma_{me}}{\Gamma_{me} + \Gamma_{ac}} - \frac{2T_{LAD}}{R_{nuc}}\right)}.$$

Thus

$$T_{LAD} = \sqrt{\frac{D_h}{\Gamma_{ac}} \frac{\Gamma_{me}}{\Gamma_{me} + \Gamma_{ac}} \frac{F\left(\frac{R_{LAD}}{l_{RD}}, \theta\right)}{\left(1 + \frac{2l_{RD}}{R_{Nuc}} F\left(\frac{R_{LAD}}{l_{RD}}, \theta\right)\right)}}.$$

On simplification

$$T_{LAD} \sim \sqrt{\frac{D_h}{\Gamma_{ac}} \frac{\Gamma_{me}}{\Gamma_{me} + \Gamma_{ac}}}\ G\left(\frac{R_{LAD}}{l_{RD}}, \theta\right). \qquad \text{(S36)}$$

Here, $G\left(\frac{R_{LAD}}{l_{RD}}, \theta\right)$ is a function that measures the total diffusive influx of methyl marks and is contingent on both the LAD-lamina contact angle θ and the LAD radius R_{LAD}/l_{RD}. The function $G\left(\frac{R_{LAD}}{l_{RD}}, \theta\right)$ can be obtained accurately numerically to obtain the dependence of LAD thickness on epigenetic rates $(\Gamma_{me}, \Gamma_{ac})$ and chromatin-lamina affinity $(V_{LAD} \sim \gamma_{he} \cos\theta)$ as shown in next section.

6 Numerical Solution for LAD Thickness

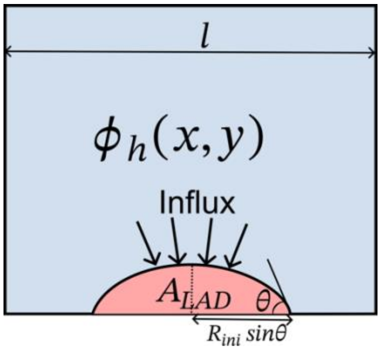

Supplementary Fig. 7: A schematic representation of geometry used to obtain LAD thickness numerically.

To get the precise dependence of LAD thickness T_{LAD} on histone methylation rate Γ_{me} and chromatin-lamina affinity relative to chromatin-chromatin interaction strength V_{LAD} ($\sim \gamma_{\text{he}} \cos\theta$), we start with a LAD domain of initial guess radius R_{ini} at a given histone methylation rate Γ_{me} and a constant LAD-lamina contact angle θ (Supplementary Fig. 7). The rate of change of the LAD size A_{LAD} is described by the equation:

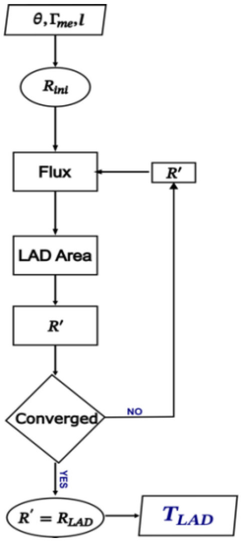

Supplementary Fig. 8: A flowchart showing the steps involved in numerical estimation of LAD thickness T_{LAD}.

$$\frac{dA_{LAD}}{dt} = D_h \nabla^2 \phi_h - (\Gamma_{ac} + \Gamma_{me})\phi_h + \Gamma_{me}. \qquad \text{(S36)}$$

We solve Eq S36 numerically for ϕ_h to determine the flux. At steady-state, the balance between influx and outflux-given $D_h \nabla^2 \phi_h$ for influx and $A_{LAD}\Gamma_{ac}$ for outflux-yields A_{LAD}. The updated LAD radius R' is calculated from A_{LAD} using: $R' = \sqrt{\frac{A_{LAD}}{\theta - 0.5 sin2\theta}}$. We then update the R_{ini} with R' and repeat the iterations (Supplementary Fig. 8) until convergence is achieved at an error threshold of 0.001. The final converged radius R_{LAD}, is used to calculate LAD thickness T_{LAD} via

$$T_{LAD} = \frac{R_{LAD}^2(\theta - \sin\theta\cos\theta)}{l}. \qquad \text{(S37)}$$

To obtain the dependence of T_{LAD} on V_{LAD} and Γ_{me}, we conducted a series of above discussed numerical simulations varying θ and Γ_{me}. The results, depicted in Supplementary Fig.10, show three distinguishable regimes in LAD thickness variation (Supplementary Fig. 10, inset) as the strength or number of chromatin anchoring proteins increases. In the next section we discuss these three regimes in detail.

7 Three regimes of LAD thickness T_{LAD} variation with chromatin-lamina affinity V_{LAD}

As discussed in Supplementary Information Section 6, the dependence of LAD thickness on chromatin-lamina affinity shows three regimes:

Regime I: Weak chromatin-lamina affinity: When the chromatin-lamina interactions at the periphery are weak, the interactions between distinctly and like-marked histones dominate, resulting in formation of bead-like peripheral heterochromatin domains with contact angles $\theta \approx 90°$ (Fig. 2D). Due to a sparse distribution of peripheral domains along the lamina, owing to low chromatin-lamina affinity, the average LAD thickness (Supplementary Fig. 9, top panel) is small. While a small increase in chromatin-lamina affinity will increase the spreading of LADs, their sparse distribution with regions of lamina not associating with chromatin results in a relatively negligible change in the LAD thickness. Indeed, as shown in Supplementary Fig. 10, within the Regime I the numerically predicted average LAD thickness does not change significantly with increase in chromatin-lamina affinity. On the other hand, as methylation rate increases (or acetylation rate decreases), there is an overall augmentation in heterochromatin content in the nucleus and at its periphery, leading to an increase in LAD thickness (Supplementary Fig. 10). Within regime I (i.e. when $\theta \sim 90°$), under dilute limit condition where LADs are very distant from each other, the discrete LADs are much smaller than the relevant reaction-diffusion length scale $R_{LAD} \ll l_{RD}$, $G\left(\frac{R_{LAD}}{l_{RD}}, \theta\right) \sim G(0,\theta)$ is a finite function of the contact angle θ, independent of R_{LAD}. In this limit, the dependence of LAD thickness on epigenetic reaction rate Γ_{me} scales as

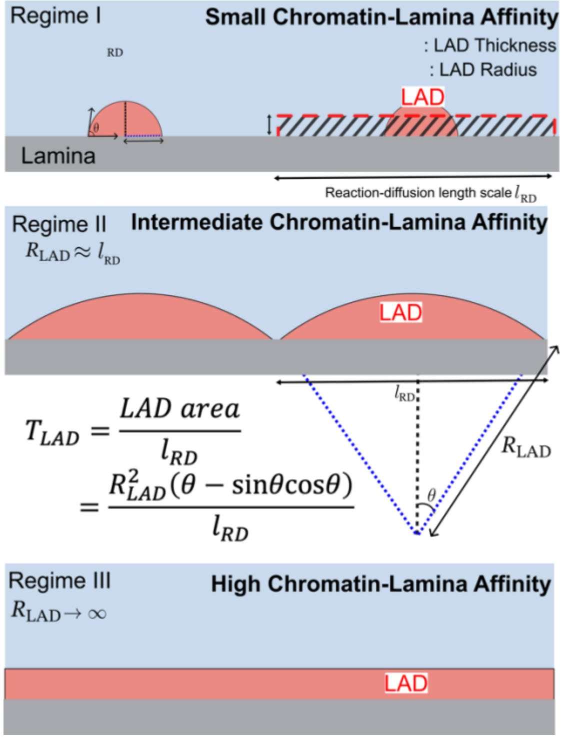

Supplementary Fig. 9: Schematics illustrating the morphology of LAD at varying levels of chromatin-lamina affinity.

$$T_{LAD} \sim \sqrt{\frac{D_h}{\Gamma_{ac}} \frac{\Gamma_{me}}{\Gamma_{ac} + \Gamma_{me}}}, \quad \text{(S37)}$$

and shows excellent agreement with the numerical solution of Eq S36 (Supplementary Fig. 11).

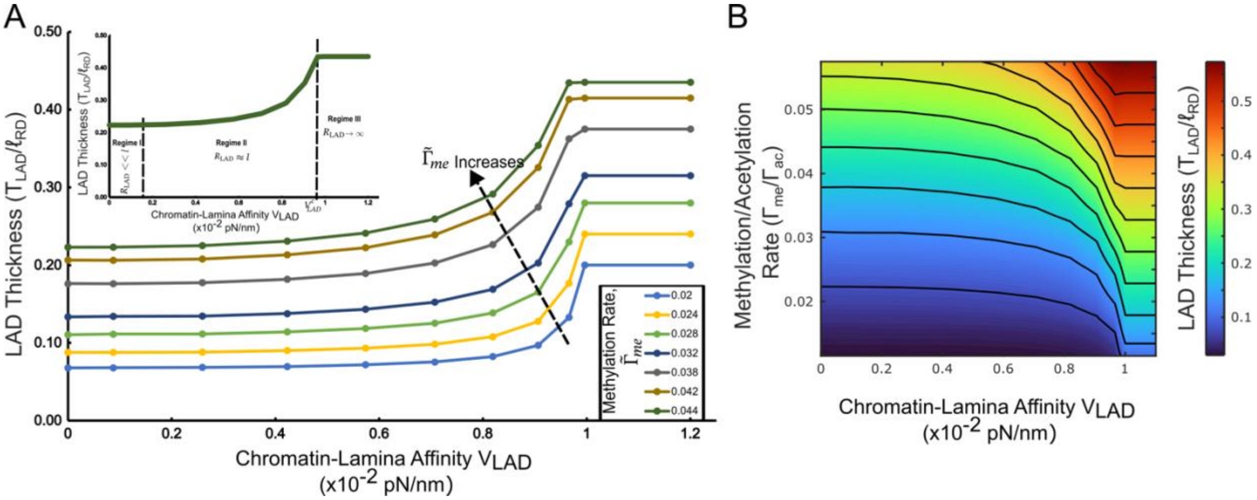

Supplementary Fig. 10: The epigenetic reaction rates $(\Gamma_{me}/\Gamma_{ac})$ and the chromatin-lamina affinity (V_{LAD}) together determine the thickness of the LADs (T_{LAD}). The numerically obtained dependance of LAD thickness on chromatin-lamina affinity and histone methylation rate. For a given V_{LAD} as methylation increases the LAD thickness increases. For a given methylation rate the LAD thickness increases with chromatin-lamina affinity. However, beyond a value of V_{LAD} any increase in methylation rate does not affect T_{LAD}. (A) The isolines shown on the phase diagram are for a constant methylation rate. The inset shows the three regimes of LAD thickness variation with chromatin-lamina affinity. (B) The isolines shown on the phase diagram are for a constant LAD thickness.

Regime II: Intermediate chromatin-lamina affinity: With increase in chromatin-lamina affinity, more chromatin is sequestered to the periphery, resulting in increased spreading of LADs along the lamina. Indeed, parametric increase in the chromatin-lamina affinity in the phase-field model shows the formation of elongated LADs (Fig. 2D) shaped like a circular segment (Supplementary Fig. 9) with radius comparable to the reaction-diffusion length scale ($R_{LAD} \sim l_{RD}$) and contact angles smaller than those in Regime I ($\theta < 90°$). The growth of LADs is also accompanied by reduced spacing between neighboring LADs (Supplementary Fig. 9). The dependence of LAD thickness on epigenetic rates and chromatin-lamina affinity in Regime II becomes non-linear (Eq S36) but can be evaluated numerically (Supplementary Fig. 10). As the chromatin-lamina affinity increases the LADs spread further, coming closer to their neighbors and reducing the LAD-free region along the lamina, thereby effectively increasing the average LAD thickness (as seen in Supplementary Fig. 10, Regime II). Moreover, we observe that increase in methylation rate increases the heterochromatin content at the periphery, thereby forming LADs of larger radius further amplifying the LAD thickness (Supplementary Fig. 10).

Regime III: High chromatin-lamina affinity: Within Regime II the elevation in chromatin-lamina affinity results in increased spreading of chromatin along the lamina bringing the neighboring LADs closer. Beyond a critical limit (V_{LAD}^c), we enter the Regime III where the LADs form a near continuous layer along the lamina (contact angle $\theta \sim 0$,Supplementary Fig. 9, lower panel). The effective radius of such LAD approaches a large value ($R_{LAD} \sim \infty$,Supplementary Fig. 9). Beyond the critical limit (V_{LAD}^c), any additional increase in the chromatin-lamina affinity alone does not alter the LAD thickness (Supplementary Fig. 10) since a continuous layer of LAD has already formed with maximal interaction between chromatin and lamina. On the other hand, as methylation rate increases (Supplementary Fig. 10) additional availability of heterochromatin increases the LAD thickness. The increased LAD thickness with increase in methylation facilitates an easier interaction between neighboring LADs, promoting the formation of a continuous layer of LADs. Hence, we observe (Supplementary Fig. 10) that with increasing methylation rates the transition

into Regime III, occurs at a lower critical limit of chromatin-lamina affinity, i.e. γ_{lh}^{c} reduces as methylation rate increases. In this regime, as contact angle $\theta \sim 0$ and $R_{LAD} \sim \infty$, consequently, $G\left(\frac{R_{LAD}}{l_{RD}}, \theta\right) \sim G(\infty, 0)$ is again independent of R_{LAD}. Thus, the dependence of thickness of LADs in Regime III on histone methylation rate Γ_{me} scales as

$$T_{LAD} \sim \sqrt{\frac{D_h}{\Gamma_{ac}} \frac{\Gamma_{me}}{\Gamma_{ac} + \Gamma_{me}}}, \quad \text{(S38)}$$

and, as in Regime I, shows excellent agreement that observed numerically (Supplementary Fig. 11).

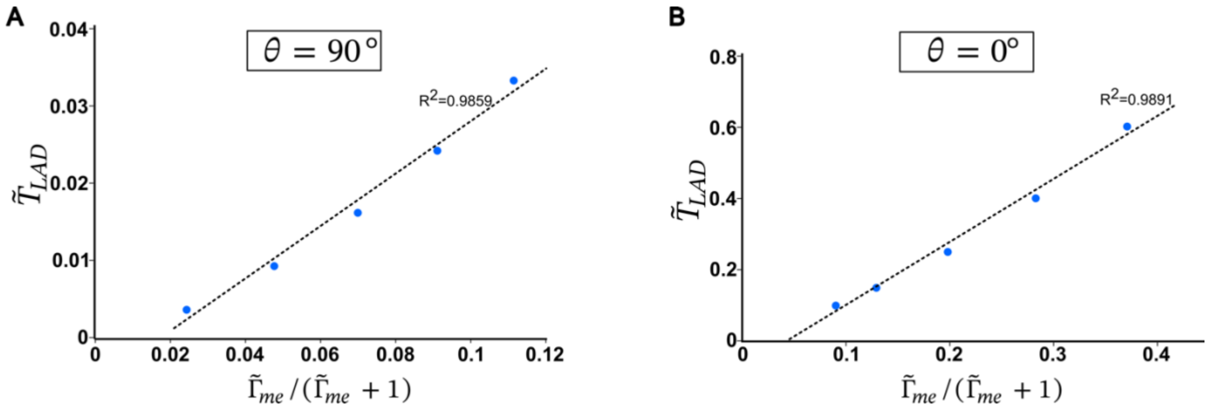

Supplementary Fig. 11: The scaling relation between T_{LAD} and methylation rate obtained numerically for (A) small chromatin-lamina affinity and (B) high chromatin-lamina affinity.

For the specific morphologies of for the flat heterochromatin layer ($\theta \sim 0°$, $R_{LAD} \sim \infty$), the LAD thickness can also be derived from the balance between the reaction-diffusion driven influx and acetylation driven outflux to obtain the scaling relations equivalent to Eq S38 as follows:

<u>Flat heterochromatin layer:</u> Here we provide the derivation for thickness scaling that directly considers the layer of heterochromatin near the border. The reaction-diffusion equation Eq. S32 can be simplified to an 1D equation,

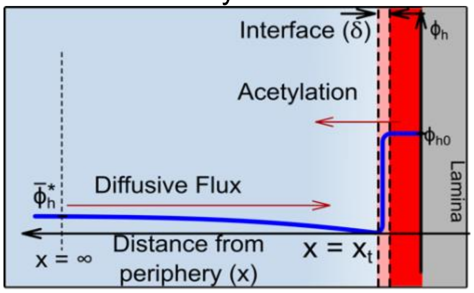

Supplementary Fig. 12: The competition of diffusion driven influx of heterochromatin with the epigenetic reaction driven outflux of heterochromatin from the LAD determines its steady state size.

$$D_h \frac{d^2\phi_h}{dx^2} - \Gamma_{ac}\phi_h + \Gamma_{me}\phi_e = 0, \quad \text{(S39)}$$

where x denotes the distance from the interface between heterochromatin and euchromatin (Supplementary Fig. 12). Two different volume fractions ϕ_h^- and ϕ_h^+ coexist immediately inside and outside the boundary of heterochromatin, respectively. Solving Eq S38 with boundary conditions $\phi_h|_{x_t^+} = \phi_h^+ = 0$ and $\phi_h|_{x=\infty} = \bar{\phi}_h^*$ gives,

$$\phi_h = -\bar{\phi}_h^* \, e^{-x/l_{RD}} + \bar{\phi}_h^*, \quad \text{(S40)}$$

where l_{RD} is the characteristic reaction-diffusion length scale given under a dilute limit as $l_{RD} = \sqrt{\frac{D_h}{\Gamma_{ac}}}$. Similar to Eq. S33, the change in the volume of the layer of heterochromatin can be written as,

$$\frac{dV}{dt} = S\frac{dT_{LAD}}{dt} = SD_h\frac{\partial \phi_h}{\partial x} - ST_{LAD}\Gamma_{ac}, \tag{S41}$$

where S denotes the surface area of the heterochromatin layer. Solving Eq. S41 at the steady state by setting $\frac{dT_{LAD}}{dt} = 0$ yields

$$T_{LAD} = \frac{D_h\bar{\phi}_h^*}{\Gamma_{ac}l_{RD}} \sim \sqrt{\frac{D_h}{\Gamma_{ac}}}\bar{\phi}_h. \tag{S42}$$

The relation in Eq. S42 agrees with Eq. S38.

On rescaling Eq S36 and S38

$$\tilde{T}_{LAD} \sim \frac{\tilde{\Gamma}_{me}}{\tilde{\Gamma}_{me} + 1}\, G(\tilde{R}_{LAD}, \theta), \tag{S4338}$$

$$\tilde{T}_{LAD} \sim \frac{\tilde{\Gamma}_{me}}{\tilde{\Gamma}_{me} + 1}. \tag{S4439}$$

8 Model parameters, Initial and boundary conditions

To capture the spatiotemporal organization of chromatin in the nucleus, we numerically solve Eq S9 and S10. We consider an initial spatially homogenous distribution of chromatin in the nucleus with a no flux boundary condition of the order parameter ($\nabla\mu_d.\hat{\boldsymbol{n}}|_{\text{boundary}} = 0$) ensuring the conservation of epigenetic marks. To mimic the intrinsic heterogeneities present in the nucleus, we add a random uniform noise to the initial chromatin configuration. In the model all length and time scales are normalized against the reaction-diffusion length scale l_{RD} and Γ_{ac} respectively. In rescaled model, the heterochromatin distribution is determined by rescaled methylation rate $\tilde{\Gamma}_{me}$ and rescaled chromatin-lamina affinity $\tilde{V}_{LAD}$. The initial values of these two parameters are chosen such that the simulation generate a distribution of HC domains comparable to the control nuclei (Supplementary Fig. 13). To represent the spatial heterogeneity of epigenetic reactions and chromatin-lamina interactions, Gaussian noise was applied to methylation reaction rates and chromatin-lamina affinity. This choice allows us to capture the key statistical features of chromatin organization while keeping the simulations computationally feasible. All initial parameters are listed in Supplementary Table 1.

Supplementary Table 1: Values of the parameters used in simulation.

	PARAMETER	DESCRIPTION	VALUE
**INITIAL CONDITIONS**	$\phi_h^{initial}$	Initial heterochromatin content in the nucleus	0.3
	$\phi_e^{initial}$	Initial euchromatin content in the nucleus	0.7

	$\phi_n^{initial}$	Initial nucleoplasm content in the nucleus	$1 - \phi_h^{initial} - \phi_e^{initial}$
	σ_ϕ^{noise}	Range of uniform perturbation due to heterogeneities in initial conditions	0.01
**EPIGENETIC KINETICS**	$\tilde{\Gamma}_{me}$	Reaction rate of histone methylation (non-dimensionalised)	0.38
	σ_Γ^{noise}	Variance in spatial distribution of reaction rates	0.2
**CHROMATIN-LAMINA INTERACTIONS**	$\tilde{V}_{LAD}$	Chromatin-lamina interaction strength (non-dimensionalised)	0.01
	$\sigma_{\tilde{V}_{LAD}}^{noise}$	Variance in spatial distribution of $\tilde{V}_{LAD}$	0.3

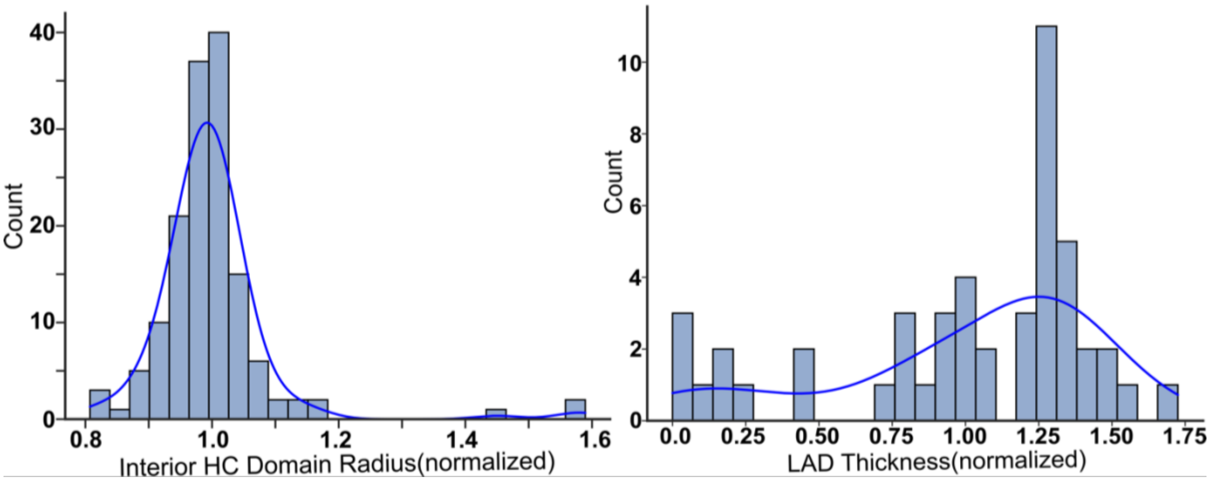

Supplementary Fig. 13: Size distribution of the heterochromatin domains obtained numerically in the interior (left) and at the periphery (right) of the control nucleus.

To simulate the change in chromatin organization in chemo-mechanical altered environments, we vary the parameter $\tilde{\Gamma}_{me}$ and $\tilde{V}_{LAD}$ according to extracted parameter from the theoretical framework (listed in Supplementary Table 2). The numerically observed change in heterochromatin domain sizes in interior and at the periphery of the nucleus (Supplementary Fig. 14) using the extracted biophysical parameters from STORM images agrees with the in-vitro observed chromatin reorganization.

While the current simulations use spatially averaged parameter values supplemented with noise, incorporating the actual inferred spatial distributions of $\tilde{\Gamma}_{me}$ and $\tilde{V}_{LAD}$ could enable finer spatial detail by directly mapping experimentally derived spatial profiles into the model. This would account for localized variations in methylation reaction rates and chromatin-lamina interactions across individual nuclei. However, such an approach would significantly increase computational complexity without significantly altering the key results, as the current method already reproduces the main experimental observations. For example, the simulated change in heterochromatin

domain sizes and LAD thicknesses align well with the in-vitro measurements obtained from STORM imaging after treatments (Supplementary Fig. S14, Supplementary Table 4).

Supplementary Table 2: Values of parameters used to simulate nuclei undergoing pharmacological and environmental interventions.

	$\tilde{\Gamma}_{me}$	$\tilde{\gamma}_{lh}$
**Control**	0.038	0.01
**GSK**	0.024	0.0103
**TSA**	0.029	0.0088
**Y27**	0.043	0.0126
**Stiff**	0.032	0.0089
**Glass**	0.030	0.0085
**Healthy Tenocytes**	0.026	0.0089

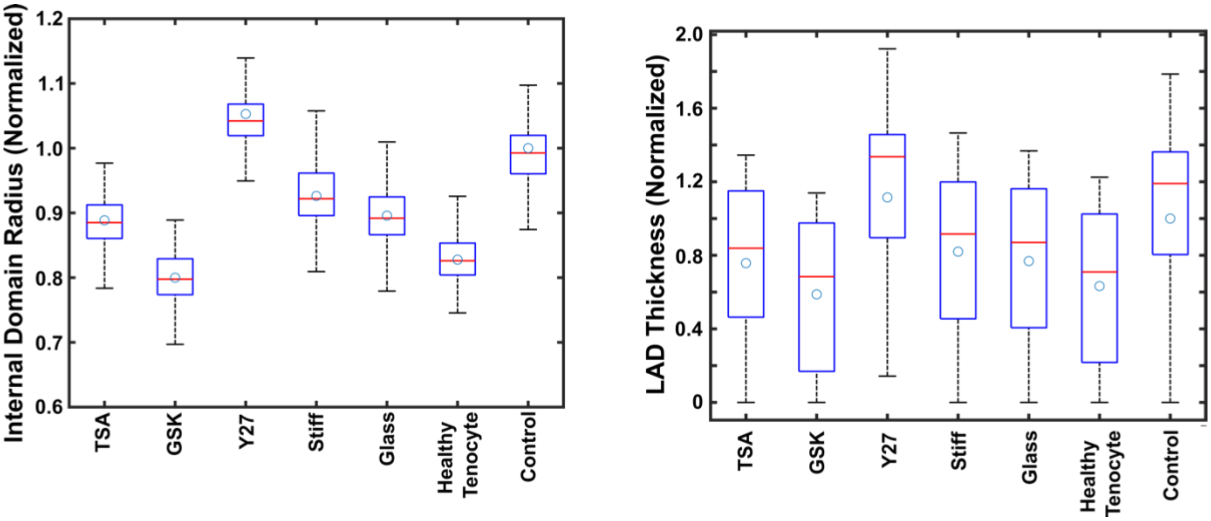

Supplementary Fig. 14: Numerical predicted change in heterochromatin domain size (A) and LAD thickness (B) in nuclei undergoing chemical-mechanical alterations.

<u>Source of heterogeneities in chromatin organization:</u> The experimentally observe heterogeneities in chromatin organization (Supplementary Fig. 17), such as variations in heterochromatin (HC) domain sizes and LAD thicknesses, can arise from multiple sources. In our framework, we account spatial heterogeneities in the availability of epigenetic factors as the primary determinants of these variations. This choice is motivated by experimental evidence and the need to adopt a minimal parameter model for biophysical interpretability. Nuclear structures such as nucleolus, or biomolecular condensates such as the transcriptional factories or the Cajal bodies, can spatially restrict the availability of epigenetic factors like histone methyltransferases and deacetylases. Our experimental measurements of EZH2 and HDAC3 distribution (Fig. 5F) show spatial variations in their local concentrations. Thus, within our framework, the epigenetic reaction parameters, which denote the availability of the epigenetic factors, are presumed to be heterogeneously distributed

spatially. These spatial heterogeneities are modeled in our framework as key parameters driving chromatin organization.

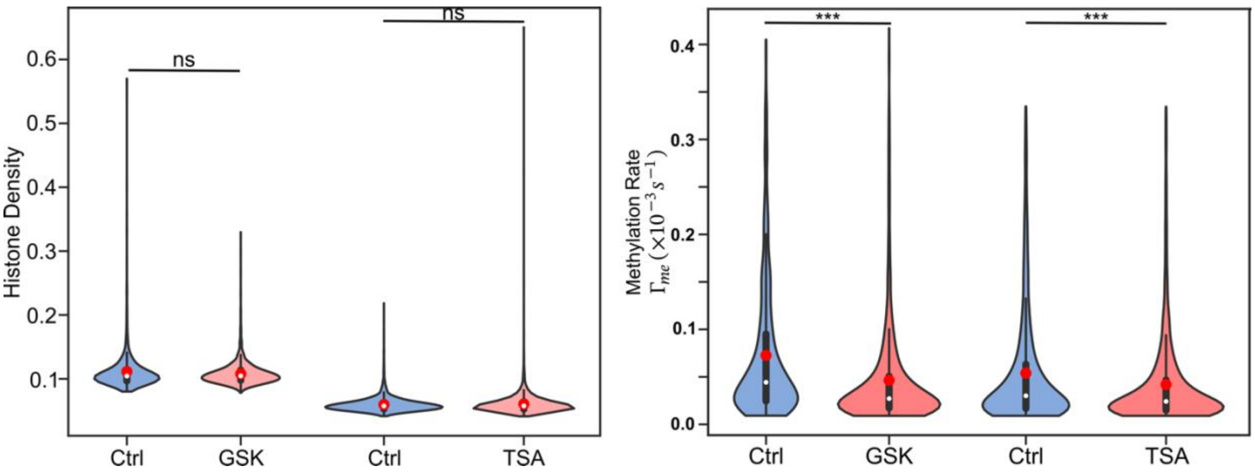

Supplementary Fig. 15: Effect of pharmacological treatments on histone density and methylation rates within heterochromatin domains. (Left) Comparison of histone density within heterochromatin domains before and after GSK (EZH2 inhibitor) and TSA (HDAC inhibitor) treatments, showing no significant spatial variation. The red dots represent the mean values for each condition. (Right) In contrast, methylation rates exhibit significant changes following GSK and TSA treatments, highlighting the strong role of epigenetic reactions in determining heterochromatin organization. Unpaired two tail test, *$p<0.05$, **$p<0.01$, ***$p<0.00$. All violin plots show a symmetric kernel density estimate (outline), the quartiles (black boxplot), the median (white dot), and the mean (red dot with line).

Pharmacological perturbations further highlight the role of epigenetic reactions in determining chromatin organization. For instance, TSA (HDAC inhibition) and GSK (EZH2 inhibition) treatments significantly alter methylation rates, which leads to measurable changes in heterochromatin domain sizes and LAD thicknesses (Supplementary Fig. 15, right panel). In contrast, we observe that histone density within HC domains does not vary significantly across these conditions(Supplementary Fig. 15, left panel), suggesting that chromatin-chromatin interaction energies—which control the compaction of nucleosomes—play a lesser role in determining HC domain size. Additionally, we numerically performed a sensitivity analysis for the methylation rate and chromatin-chromatin interaction energies, increasing each parameter by 10%, which shows that heterochromatin domain sizes are far more sensitive to changes in the methylation rate than to variations in chromatin-chromatin interactions (Supplementary Fig. 16).

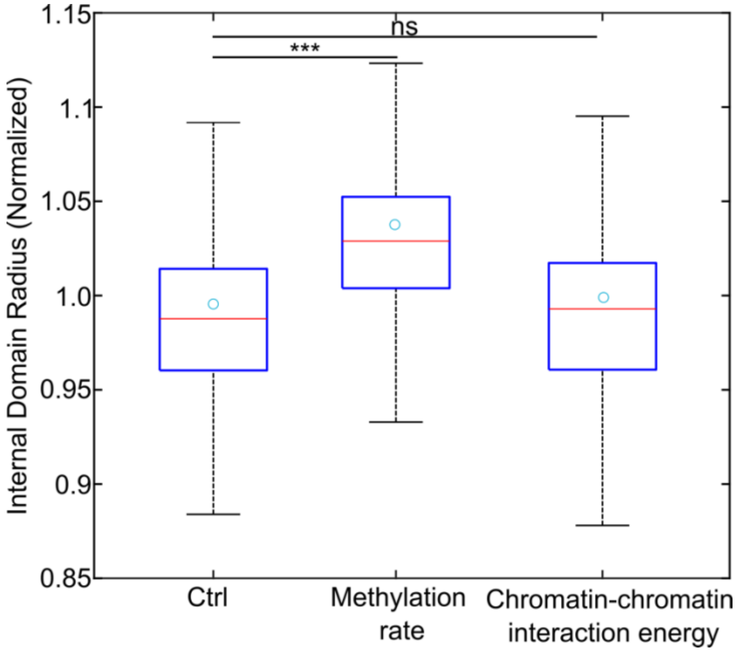

Supplementary Fig. 16: Sensitivity analysis of heterochromatin domain size to model parameters. Numerical simulations showing the relative impact of changes in methylation rate and chromatin-chromatin interaction energies on heterochromatin domain size. A 10% increase in methylation rate significantly alters the domain size, while a similar change in chromatin-chromatin interaction energies has a negligible effect. This demonstrates that heterochromatin domain sizes are far more sensitive to epigenetic reaction rates than to chromatin interaction strengths.

Note that the other model parameters still are important for determining chromatin organization, and downstream gene expression. However, when it comes to quantitative STORM image analysis – to extract the size scale of heterochromatin domains – we find that the other parameters such as chromatin-chromatin interaction energies are not immediately relevant.

9 Physiological values of model parameters

The numerical simulations are performed with rescaled parameters as discussed in Supplementary Information Section 1.5. The rescaling of the mathematical model is done with respect to the intrinsic energy scale (chromatin-chromatin interaction strength), time-scale (reaction rates) and length-scale (reaction-diffusion length). To physically interpret our simulation results, the physiological values of these model parameters are evaluated as listed in Supplementary Table 3.

Supplementary Table 3: Value of non-dimensional parameters in physical dimensions

	PARAMETER	DESCRIPTION	VALUE	REFERENCE
**ENERGY SCALE**	$c \sim \left(\frac{2-4k_BT}{V_N}\right)$	Scale of chromatin-chromatin interactions	$\sim 8 \times 10^{-3} - 16 \times 10^{-3} pN/nm^2$	[7]
	l_{int}	Interface width of heterochromatin domains	$10 - 50\ nm$ Observed from ChromSTEM imageing	[3]

	$\kappa = (l_{int}^2 \times c)$	the penalty associated with the interface formation	$\sim 0.8pN$	
	$\gamma = \left(\frac{1}{6}\sqrt{\kappa c}\right)$	Surface tension	$\sim 10^{-2} pN/nm$	
	$V_{LAD}^{max} = (\gamma cos\theta)$	Maximum strength of chromatin-lamina interactions per unit area	$\sim 10^{-2} pN/nm$	[8]
**TIME SCALE**	Γ_{ac}	Reaction rate of histone acetylation	$\sim 10^{-2}\ s^{-1}$	[9]
	Γ_{me}	Reaction rate of histone methylation	$\sim 10^{-3}\ s^{-1}$	[10]
**LENGTH SCALE**	D	Diffusivity of nucleosome	$\sim 10^{-3}\ \mu m^2/s$	[11]
	$l_{RD} \sim \sqrt{\frac{D}{\Gamma_{ac}}}$	Reaction-diffusion length scale	$\sim 300\ nm$	

10 Example of extraction process of biophysical parameters in control nuclei from STORM images using theory

Here, we present the extraction process for hMSCs nuclei cultured on soft hydrogel under control conditions. We start by generating H2B density heatmaps from segmented super-resolution images using Voronoi tessellation (Supplementary Fig. 17 top panel). These heatmaps show the spatial variability in chromatin distribution, including regions of low heterochromatin density that may correspond to nucleoli, RNA-rich compartments, or other nuclear substructures, as noted in Supplementary Fig. 17. Such regions are typically low in heterochromatin and contribute to the observed heterogeneity. Additionally, certain technical factors, such as slight out-of-focus imaging, may amplify this variability.

The processed images are analyzed using a custom MATLAB code (section 4.4)[12] to obtain the statistical distributions of the interior domain radii R_d and the LAD thickness T_{LAD} for each nucleus (Supplementary Fig. 17 middle panel). The derived interior domain and LAD sizes are locally correlated with the methylation rate Γ_{me} via Eqs S19 and S25, enabling the extraction of nucleus-wide distribution of Γ_{me} (Supplementary Fig. 17 bottom panel). Finally, leveraging the extracted distributions of Γ_{me} and LAD thickness, we apply Eq S36 (numerically depicted in Supplementary Fig. 10) to infer the distribution of chromatin-lamina affinity relative to chromatin-chromatin interaction strength V_{LAD} along the nuclear periphery (Supplementary Fig. 17 bottom panel). Notably, the distributions of extracted parameters in individual nuclei feature characteristic means (red dot) that are comparable to the mean across all nuclei (blue dot).

In a similar fashion, we extract the distribution of biophysical parameters for all nuclei undergoing known pharmacological treatments and biomechanical stimuli. The quantitative changes in the mean values of these parameters, relative to the mean values of control nuclei for each treatment, are listed in Supplementary Table 4. The general trend observed from Supplementary Table 4

indicates that a decrease (or increase) in the methylation rate Γ_{me} is accompanied by a corresponding decrease (or increase) in the interior domain radii R_d. This aligns with our theoretical predictions in Eq S19 which evaluates the size of heterochromatin domains based on the kinetic balance between methylation-diffusion driven heterochromatin influx and acetylation driven euchromatin outflux.

However, as discussed in Supplementary Information Section 4 (Eq S25), our theory also predicts that increased chromatin-lamina interactions can lead to substantial sequestering of chromatin towards the periphery, reducing the heterochromatin available in the nucleus interior. In such a scenario, even with increased methylation, some heterochromatin may move towards the periphery, resulting in reduction of interior heterochromatin domain sizes.

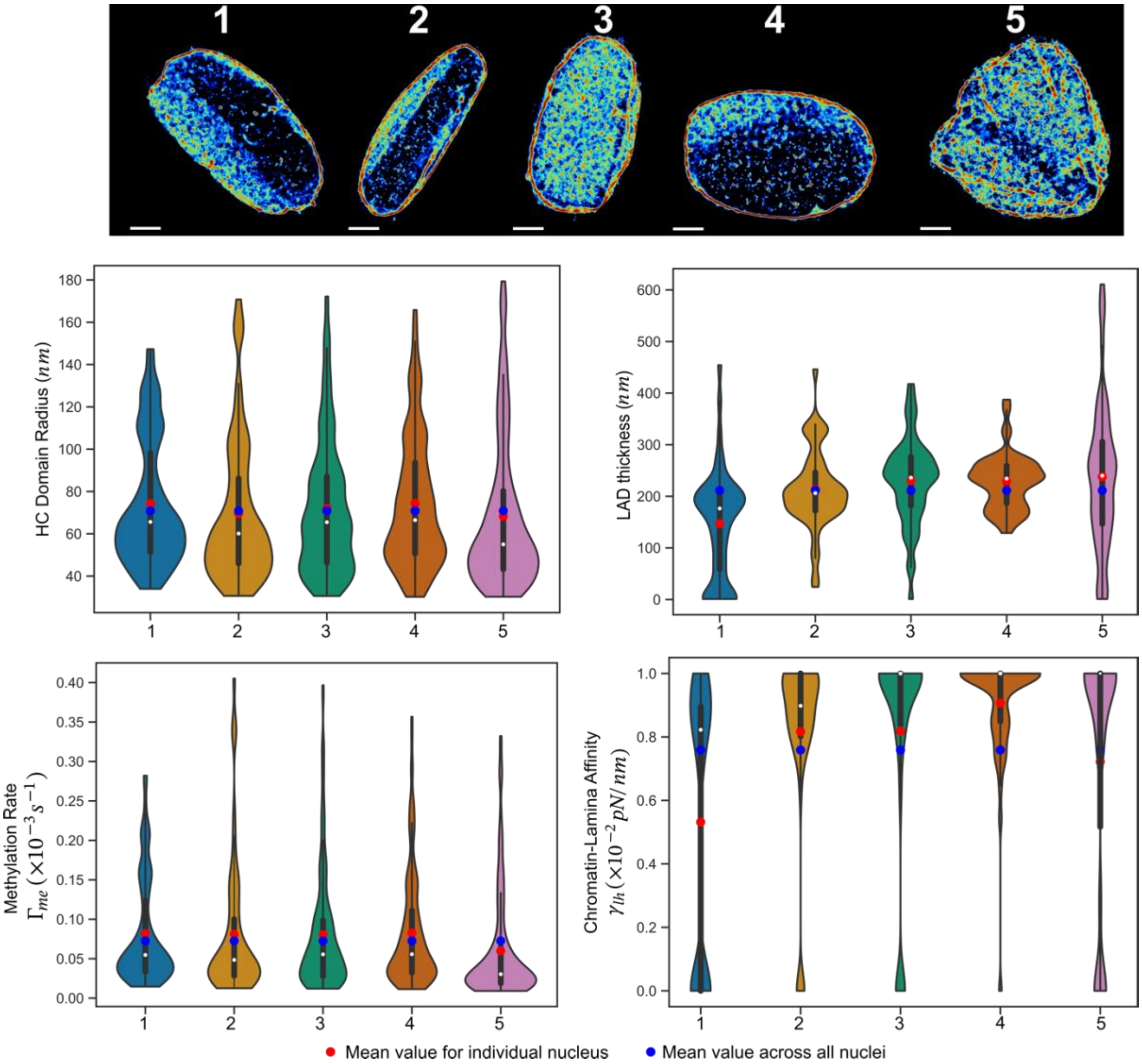

Supplementary Fig. 17: Top Panel: Voronoi density plots of H2B STORM images of hMSCs cultured on soft hydrogel nuclei cultured in control condition. Middle Panel: Quantification of STORM images showing distribution of domain radius and LAD thickness for individual nucleus. Bottom Panel: Distribution of histone methylation rate and chromatin-lamina affinity obtained by our integrative framework. All violin plots show a symmetric kernel density estimate (outline), the quartiles (black boxplot), the median (white dot), and the mean (red dot with line).

Supplementary Table 4: Change in biophysical parameters relative to control nuclei

	QUNATIFIED FROM STORM IMAGES		PREDICTED BY INTERGATIVEFRAMEWORK		PREDICATED BY SIMULATIONS	
	R_d	T_{LAD}	Γ_{me}	V_{LAD}	R_d	T_{LAD}
**GSK**	18% (↓)	20% (↓)	36% (↓)	-	20% (↓)	30% (↓)
**TSA**	11% (↓)	26% (↓)	22% (↓)	11% (↓)	11% (↓)	22% (↓)
**Y27**	4.3% (↓)	19% (↑)	12% (↑)	26% (↑)	7% (↑)	15% (↑)
**STIFF**	6.8% (↓)	14% (↓)	17 % (↓)	10 % (↓)	7% (↓)	15% (↓)
**GLASS**	8% (↓)	21.5% (↓)	19.9% (↓)	14.5 % (↓)	10% (↓)	25% (↓)
**TENDINOSIS**	5.8% (↑)	10.2% (↑)	30% (↑)	11.6% (↑)	16% (↑)	28% (↑)

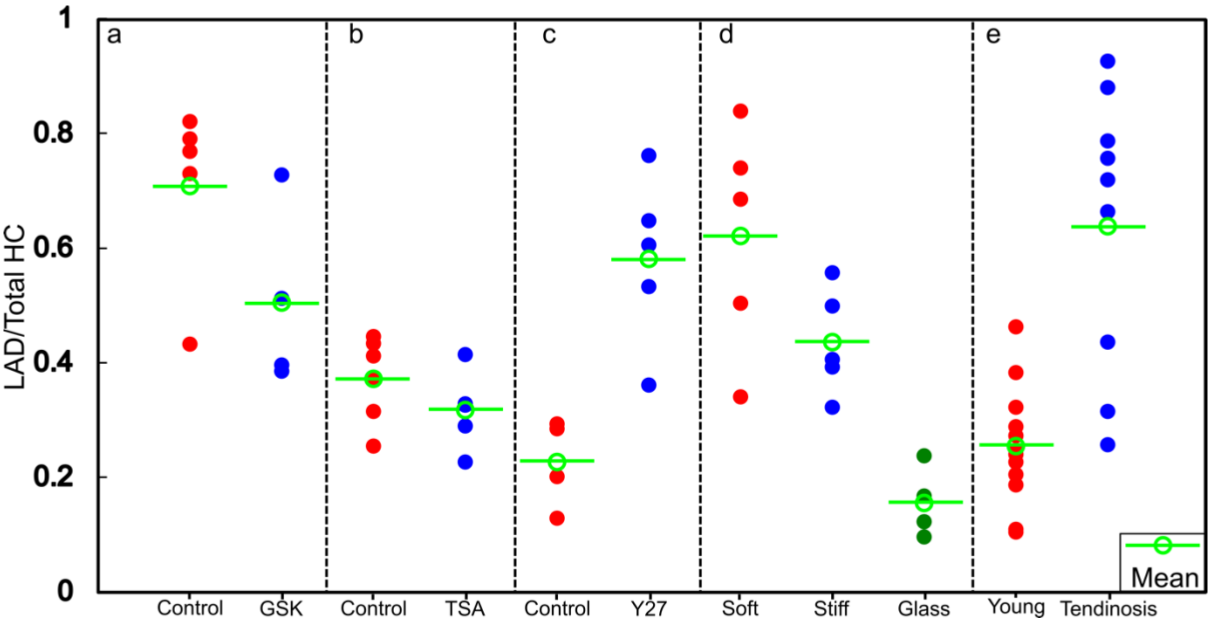

Supplementary Fig. 18: The quantification of heterochromatin localization to periphery relative to total heterochromatin in nucleus undergoing different treatments and control of each case (red plots).

This effect is observed in the case of Y27 treatment where methylation increases by 12% (Supplementary Table 4), but chromatin-lamina affinity increases more significantly (by 26%), resulting in a slight (4.3%) reduction in the mean size of the interior domains. To confirm the significant sequestering of heterochromatin towards the periphery, we plot the ratio of peripheral heterochromatin to total heterochromatin for each nucleus after control and Y27 treatment (Supplementary Fig. 18c). Indeed, there was a significant increase in the relative amount of heterochromatin at the periphery after Y27 treatment.

Interestingly, similar sequestering of heterochromatin from nucleus interior to the periphery is also observed in some other treatments viz. on soft substrates (Supplementary Fig. 18d), after tendinosis (Supplementary Fig. 18e) and in the control case of GSK treatment (Supplementary Fig. 18a). However, Supplementary Table 4 shows that in these cases the increase in chromatin-

lamina affinity (which promotes chromatin sequestering to the lamina) was countered by the increase in methylation rate (which enlarges the interior domains). Thus, a balance between chromatin sequestering and histone methylation rate regulates interior heterochromatin domain sizes.

It is important to note that in all treatments, the effect of chromatin sequestering cannot be overlooked. For example, a 36% change in methylation rate (GSK treatment) results in 18% change in domain sizes when the chromatin lamina affinity is unchanged. However, after tendinosis a 30% change in methylation rate only resulted in 5.8% change in domain sizes. This is because tendinosis also resulted in 11.6% increase in chromatin-lamina affinity causing some heterochromatin to move towards the periphery thereby limiting the growth of interior domains.

11 Procedure for Identification of Lamina Associated Domains from STORM Images

As discussed in Section 4.4, MATLAB was used for the analysis of STORM images, with chromatin density quantified via Voronoi tessellation-based segmentation. Heterochromatin domains were identified using the Density-Based Spatial Clustering of Applications with Noise (DBSCAN) algorithm, grouping neighboring Voronoi polygons into distinct clusters.

To distinguish lamina-associated domains (LADs) from interior heterochromatin, we classified a cluster as a LAD if any localization fell within a predefined threshold distance from the nuclear envelope. This threshold was set at 2.5% of the nuclear radius, based on the approximate size and localization of nuclear lamina proteins and their interaction zones. Lamina-associated proteins, such as Lamin B1 and associated chromatin interactions typically localize within a region of ~100–300 nm from the nuclear envelope [13, 14]. For nuclei with radii ranging from 5000-9000 nm, this corresponds to approximately 2-3% of the nuclear radius, making 2.5% threshold biologically relevant.

To ensure the robustness of this threshold, we had performed sensitivity analyses by testing slightly lower and higher thresholds for which data was not shown. These tests showed that the key features of LAD organization and the inferred parameters (Γ_{me} and V_{LAD}) alter insignificantly within this range (Supplementary Fig. 19), confirming the robustness of our conclusions.

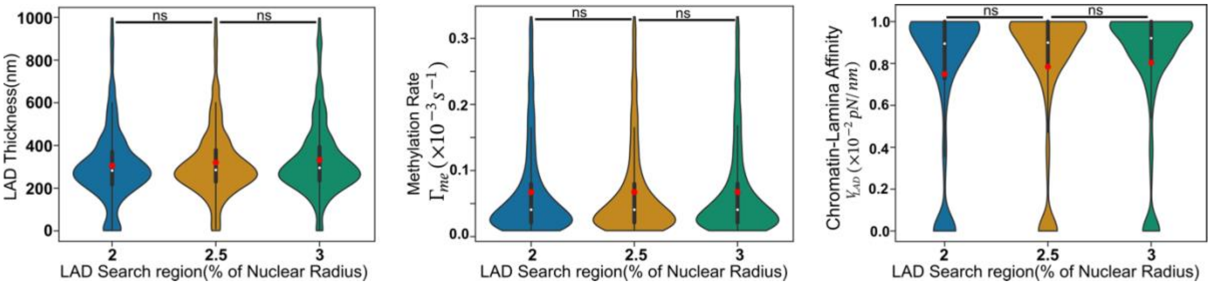

Supplementary Fig. 19: Sensitivity analysis of LAD parameters to the search region threshold. Violin plots showing the distribution of LAD thickness (left), methylation rate (Γ_{me}, middle), and chromatin-lamina affinity (V_{LAD},right) for varying LAD search region thresholds (2.0%, 2.5%, and 3.0% of the nuclear radius). Unpaired two tail test, *p<0.05, **p<0.01, ***p<0.00. All violin plots show a symmetric kernel density estimate (outline), the quartiles (black boxplot), the median (white dot), and the mean (red dot with line).

However, thresholds that were too low failed to capture chromatin regions near the lamina that visually correspond to LADs, while thresholds that were too high began misclassifying some interior chromatin domains as LADs (Supplementary Fig. 20). This analysis confirmed that the 2.5% threshold strikes an optimal balance, accurately capturing LADs while avoiding misclassification of interior domains (Supplementary Fig. 20).

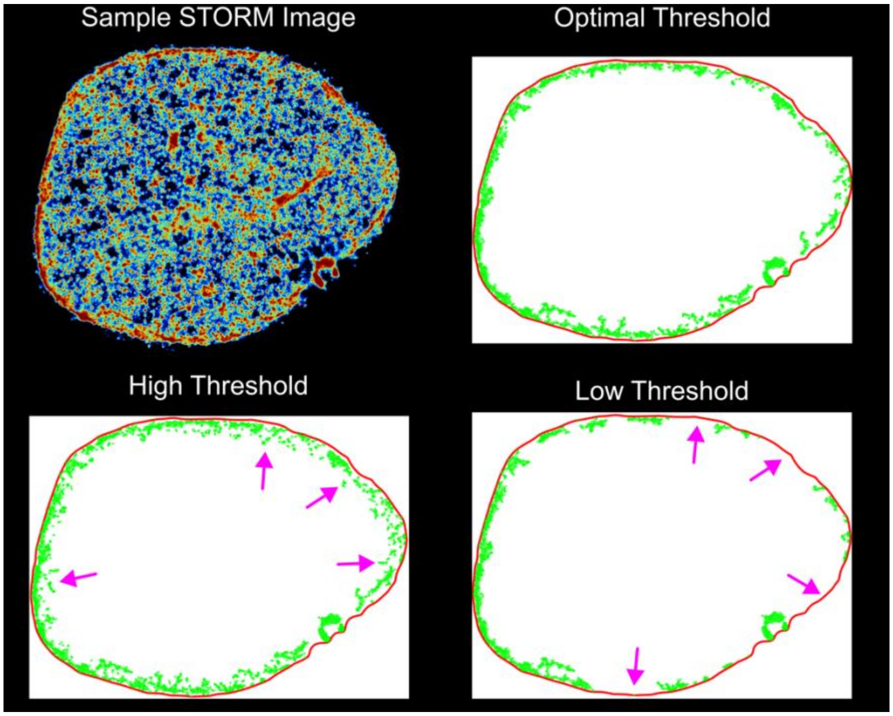

Supplementary Fig. 20: The Figure shows the impact of varying thresholds on LAD identification, with the 2.5% threshold (optimal) accurately capturing LADs, while higher or lower thresholds lead to misclassification (pink arrows) or missed (pink arrows) LADs (bottom row).

Additionally, we visually inspected the STORM images and confirmed that LAD regions identified using the 2.5% threshold closely align with chromatin regions expected to be associated with the nuclear lamina (Supplementary Fig. 20).

12 Comparison of Predicted Chromatin-Lamina Affinity Spectrum with LaminB1 ChIP-seq Data

As discussed in Section 2.4 of the main manuscript, the distribution of chromatin-lamina affinity V_{LAD} predicted by our framework exhibits a bimodal pattern, with distinct peaks corresponding to very low and very high chromatin-lamina affinity. These peaks correspond to specific nuclear regions: (1) very low-affinity regions corresponding to nuclear periphery areas deficient in chromatin-binding proteins such as nuclear pores, and (2) high-affinity regions representing strong chromatin-lamina anchoring mediated by chromatin-binding proteins. Between these two peaks, we identified a spectrum of intermediate chromatin-lamina affinities, indicative of weaker but nonzero interactions. This predicted bimodal distribution of chromatin-lamina affinity aligns qualitatively with the classification of LADs in Lamin B1 ChIP-seq data into Type 1 (T1) LADs and Type 2 (T2) LADs. Specifically:

- **T1 LADs** exhibit strong, stable interactions with Lamin B1, consistent with the peak of high V_{LAD} observed in our framework. These LADs correspond to regions of highly compacted chromatin, characterized by strong transcriptional repression and low chromatin accessibility.
- **T2 LADs** represent regions with weaker Lamin B1 association and higher chromatin accessibility. These LADs correspond to the spectrum of low-to-intermediate V_{LAD} values observed in our model.

While both LB1 ChIP-seq and our STORM-based framework consistently identify regions of strong and weak chromatin-lamina interactions, their methodologies and interpretations differ significantly (Supplementary Fig. 21 A). LB1 ChIP-seq maps genome-wide chromatin-lamina interactions by determining the likelihood of specific genomic regions being attached to Lamin B1 over a large cell population. In contrast, our STORM-based framework provides a single-cell snapshot of LAD organization, capturing spatial heterogeneity and dynamic, probabilistic interactions at the moment of cell fixation. For example, T1 LADs identified by LB1 ChIP-seq are nearly always attached to the lamina (attachment probability ~1), whereas T2 LADs are more dynamic and attach with lower probability (say ~0.25). In a single-cell snapshot, our framework observes only the LADs physically attached at the time of fixation. Thus, we may observe most of the T1 LADs (high-affinity) but only about 25% of the T2 LADs identified by ChIP-seq. This results in fewer observed T2 LADs, reflecting their weaker interaction strengths and lower attachment probabilities (Supplementary Fig. 21 B).

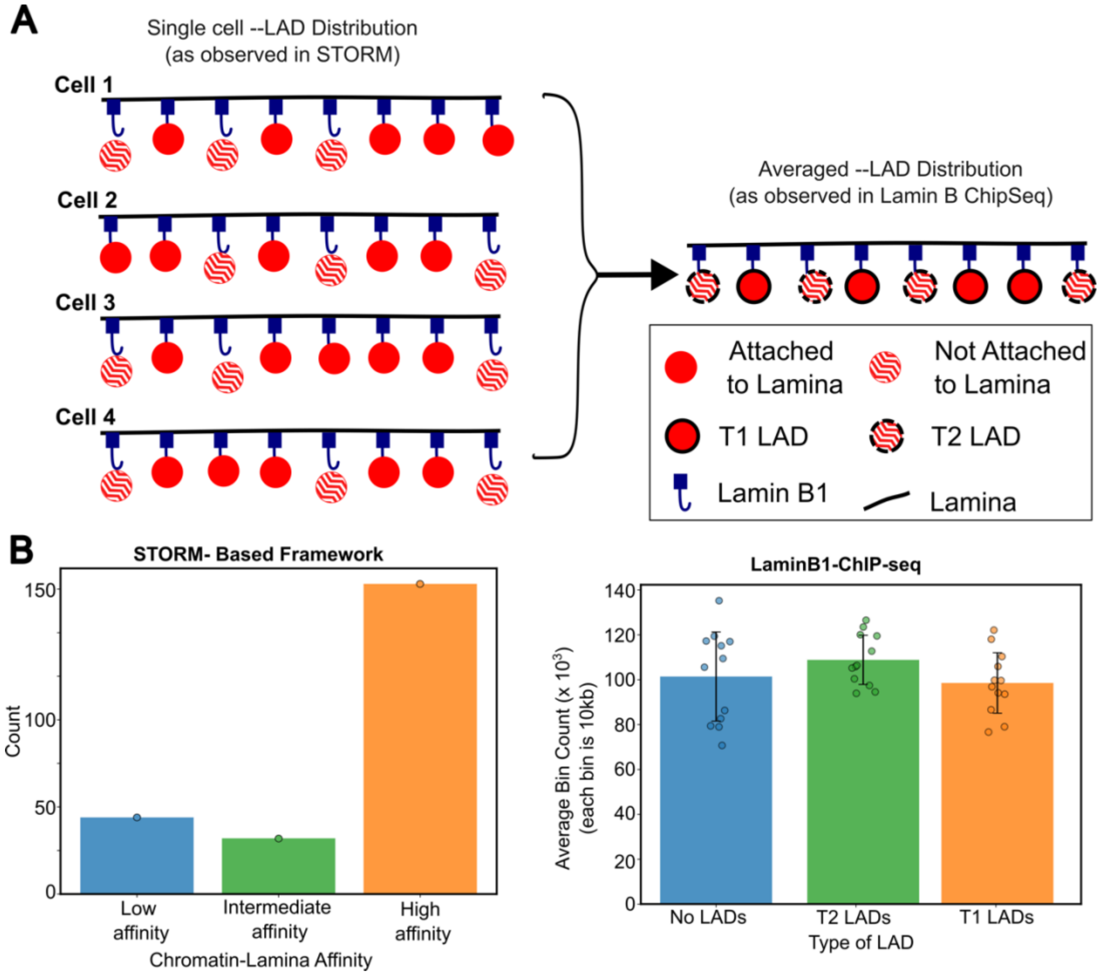

Supplementary Fig. 21: Comparison of chromatin-lamina interactions as observed in STORM-based single-cell analysis versus LB1 ChIP-seq (A) Schematics showing how LB1 ChIP-seq measures averaged chromatin–lamina interactions across a population of cells while the STORM-based framework captures single-cell, spatially resolved snapshots of LADs physically tethered to the lamina at fixation. (B) Quantification showing that T1 LADs are frequently detected in STORM imaging due to their high attachment probability, whereas only a subset of T2 LADs is observed per cell, consistent with their dynamic, probabilistic lamina associations.

Additionally, the two methods differ in the type of information they provide. LB1 ChIP-seq identifies “where” along the chromatin polymer are the LADs located but does not resolve their 3D spatial arrangement within the nucleus. In contrast, our framework reveals “how” LADs are organized

spatially, visualizing the spatial clusters of the nucleosomes along the nuclear lamina, identifying the physics governing the size scaling of these clusters, and as well as the variabilities across cells. Together, these methods provide complementary insights: LB1 ChIP-seq identifies which genomic regions interact with Lamin B1, while our framework reveals how these interactions occur spatiotemporally at the single-cell level. This integration of genomic and biophysical perspectives provides a more complete understanding of chromatin-lamina interactions and their role in nuclear architecture.

By integrating the two sets of data from single-cell modeling and population-averaged sequencing – both of which have their advantages – a more holistic understanding of spatiotemporal LAD organization can be achieved. One promising future direction involves using probabilistic modeling to integrate our framework predictions with population-level ChIP-seq data, allowing us to compute the "chromatin-lamina attachment probabilities" for specific LAD regions in individual cells. The "chromatin-lamina attachment probability" is the probability that a specific LAD region is associated with the nuclear lamina (say via lamin B1) in an individual cell.

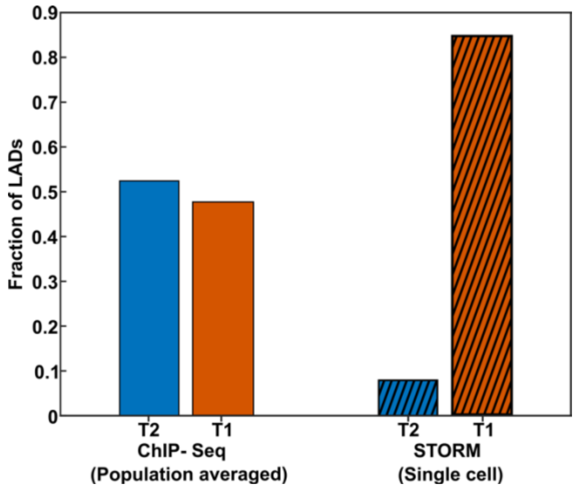

Supplementary Fig. 22: The fraction of LADs of type T1 and T2 based on population-averaged ChIP-seq data (solid color bars, left side) and single-cell STORM-based data (hatched bars, right side).

For instance, let us consider the lamin B1-chromatin interaction data available from laminB1-ChIPSeq [15]. The population-averaged experimental data demonstrates the presence of T1 LADs with strong and T2 LADs with weaker chromatin-lamina interactions [15]. From the ChIP-seq data [15], which is averaged over a large population ($\sim 10^6$ cells) we see that nearly 47% of LADs are of type T1 while the remaining (53%) are T2 type LADs, as visualized in Supplementary Fig. 22 (solid color bars, left side). In this large population, if we take a single cell, the probability that a T1 LAD is associated with lamin B1 in that specific cell is P_{T1}, while that for a T2 LAD is P_{T2}.

Our single cell STORM imaging based computational framework can be used to extract the values of these probabilities for the imaged cell when used together with LB1-ChIP-seq data. For the specific cell (Supplementary Fig. 17 number 4) in Supplementary Fig. 22, by analyzing the STORM image using our theory, we find that nearly 85% of peripheral chromatin has a very strong chromatin-lamina interaction strength, while nearly 8% demonstrates a weaker chromatin-lamina interaction strength. Comparing these percentages with the data from LB1-ChIP-seq for this specific cell, we find that the attachment probabilities of $P_{T1} = 0.85$ and $P_{T2} = 0.15$ will give the distribution of chromatin-lamina affinity observed using the framework, as shown in Supplementary Fig. 22 (hatched bars, right side). To generalize this approach, we can construct a probability distribution of chromatin-lamina interactions across multiple single-cell STORM images and compare it to ChIP-seq enrichment scores at corresponding genomic loci and quantify the likelihood that a genomic region identified as a LAD in ChIP-seq is also observed as attached in single-cell imaging. This integration enables us to assess heterogeneity in LAD attachment across cells, revealing population-level trends that would be missed by either method alone.

Further refinements of this approach could incorporate STORM imaging of specific epigenetic marks (e.g., H3K9me3 and H3K27me3) to explore correlations between heterochromatin states (facultative vs. constitutive) and chromatin-lamina attachment probabilities. In this way our framework can integrate theoretical modeling, genomic sequencing, and single-cell super-

resolution imaging, ultimately bridging population-scale genomic data with single-cell nuclear architecture for a more holistic understanding of nuclear organization.

Supplementary References

1. Briand, N. and P. Collas, *Lamina-associated domains: peripheral matters and internal affairs.* Genome Biol, 2020. **21**(1): p. 85.
2. Kiseleva, A.A. and A. Poleshko, *The secret life of chromatin tethers.* FEBS Lett, 2023. **597**(22): p. 2782-2790.
3. Kant, A., et al., *Active transcription and epigenetic reactions synergistically regulate meso-scale genomic organization.* Nature communications, 2024. **15**(1): p. 4338.
4. Sanulli, S., et al., *HP1 reshapes nucleosome core to promote phase separation of heterochromatin.* Nature, 2019. **575**(7782): p. 390-394.
5. Manzo, S.G., L. Dauban, and B. van Steensel, *Lamina-associated domains: Tethers and looseners.* Curr Opin Cell Biol, 2022. **74**: p. 80-87.
6. Harr, J.C., A. Gonzalez‐Sandoval, and S.M. Gasser, *Histones and histone modifications in perinuclear chromatin anchoring: from yeast to man.* EMBO reports, 2016. **17**(2): p. 139-155.
7. Moller, J., J. Lequieu, and J.J. de Pablo, *The free energy landscape of internucleosome interactions and its relation to chromatin fiber structure.* ACS central science, 2019. **5**(2): p. 341-348.
8. Tolokh, I.S., et al., *Strong interactions between highly dynamic lamina-associated domains and the nuclear envelope stabilize the 3D architecture of Drosophila interphase chromatin.* Epigenetics Chromatin, 2023. **16**(1): p. 21.
9. Waterborg, J.H., *Dynamics of histone acetylation in vivo. A function for acetylation turnover?* Biochemistry and cell biology, 2002. **80**(3): p. 363-378.
10. Haws, S.A., et al., *Intrinsic catalytic properties of histone H3 lysine-9 methyltransferases preserve monomethylation levels under low S-adenosylmethionine.* Journal of Biological Chemistry, 2023. **299**(7).
11. Nozaki, T., et al., *Condensed but liquid-like domain organization of active chromatin regions in living human cells.* Science Advances, 2023. **9**(14): p. eadf1488.
12. Heo, S.-J., et al., *Aberrant chromatin reorganization in cells from diseased fibrous connective tissue in response to altered chemomechanical cues.* Nature biomedical engineering, 2023. **7**(2): p. 177-191.
13. Kind, J., et al., *Single-cell dynamics of genome-nuclear lamina interactions.* Cell, 2013. **153**(1): p. 178-192.
14. Nmezi, B., et al., *Concentric organization of A- and B-type lamins predicts their distinct roles in the spatial organization and stability of the nuclear lamina.* Proc Natl Acad Sci U S A, 2019. **116**(10): p. 4307-4315.
15. Shah, P.P., et al., *An atlas of lamina-associated chromatin across twelve human cell types reveals an intermediate chromatin subtype.* Genome Biol, 2023. **24**(1): p. 16.
