# Supplementary material for: Revealing the biophysics of lamina-associated domain formation by integrating theoretical modeling and high-resolution imaging
Source: Nat Commun. 2025 Aug 25;16:7909. doi: 10.1038/s41467-025-63244-1 (PMC12378204; doi:10.1038/s41467-025-63244-1)
Supplement: Supplementary file 3 — Reporting Summary [file 41467_2025_63244_MOESM3_ESM.pdf]

nature portfolio

Corresponding author(s): Vivek B. Shenoy

Last updated by author(s): July 16,2025

Reporting Summary

Nature Portfolio wishes to improve the reproducibility of the work that we publish. This form provides structure for consistency and transparency in reporting. For further information on Nature Portfolio policies, see our Editorial Policies and the Editorial Policy Checklist.

Statistics

For all statistical analyses, confirm that the following items are present in the figure legend, table legend, main text, or Methods section.

n/a	Confirmed	
☐	☒	The exact sample size (*n*) for each experimental group/condition, given as a discrete number and unit of measurement
☐	☒	A statement on whether measurements were taken from distinct samples or whether the same sample was measured repeatedly
☐	☒	The statistical test(s) used AND whether they are one- or two-sided<br>*Only common tests should be described solely by name; describe more complex techniques in the Methods section.*
☒	☐	A description of all covariates tested
☒	☐	A description of any assumptions or corrections, such as tests of normality and adjustment for multiple comparisons
☐	☒	A full description of the statistical parameters including central tendency (e.g. means) or other basic estimates (e.g. regression coefficient) AND variation (e.g. standard deviation) or associated estimates of uncertainty (e.g. confidence intervals)
☐	☒	For null hypothesis testing, the test statistic (e.g. *F*, *t*, *r*) with confidence intervals, effect sizes, degrees of freedom and *P* value noted<br>*Give P values as exact values whenever suitable.*
☒	☐	For Bayesian analysis, information on the choice of priors and Markov chain Monte Carlo settings
☒	☐	For hierarchical and complex designs, identification of the appropriate level for tests and full reporting of outcomes
☒	☐	Estimates of effect sizes (e.g. Cohen's *d*, Pearson's *r*), indicating how they were calculated

Our web collection on statistics for biologists contains articles on many of the points above.

Software and code

Policy information about availability of computer code

Data collection	Collection of STORM image datasets is originally described in Otterstrom et al., Nucleic Acid Res 2019 (TSA Treatment) and Heo et al.,Nat. Biomed. Eng 2023 .<br>Collection of confocal Airyscan image datasets is obtained from Zeiss LSM900 airyscan platform.
Data analysis	STORM images were analyzed using a custom written MATLAB code (made publicly available and citable on GitHub at: https://github.com/ShenoyLab/STORM_Analysis_Parameter_Extraction) to obtain the sizes of the heterochromatin domains.<br><br>PDEs describing the mathematical model were solved using COMSOL, a commercially available software, using weak form PDE module.<br><br>Softwares used for analysis: MATLAB R2022a (STORM domain analysis); Image J/FIJI (Post processing of confocal Airyscan images); Microsoft Excel (for statistical analysis).

For manuscripts utilizing custom algorithms or software that are central to the research but not yet described in published literature, software must be made available to editors and reviewers. We strongly encourage code deposition in a community repository (e.g. GitHub). See the Nature Portfolio guidelines for submitting code & software for further information.

Data

Policy information about availability of data

All manuscripts must include a data availability statement. This statement should provide the following information, where applicable:

- Accession codes, unique identifiers, or web links for publicly available datasets
- A description of any restrictions on data availability
- For clinical datasets or third party data, please ensure that the statement adheres to our policy

The authors declare that the data supporting the findings of this study are available within the paper and its supplementary information files. The data generated in this study are provided in Source Data file.

Research involving human participants, their data, or biological material

Policy information about studies with human participants or human data. See also policy information about sex, gender (identity/presentation), and sexual orientation and race, ethnicity and racism.

Reporting on sex and gender	Primary human cells from three de-identified donors (two males aged 23 and 22 years, one female aged 18 years).
Reporting on race, ethnicity, or other socially relevant groupings	Not provided by vendor
Population characteristics	Commercially obtained primary human cells (Lonza).
Recruitment	Commercial purchase from Lonza; no direct recruitment
Ethics oversight	Not applicable for commercially obatined cells.

Note that full information on the approval of the study protocol must also be provided in the manuscript.

Field-specific reporting

Please select the one below that is the best fit for your research. If you are not sure, read the appropriate sections before making your selection.

- [x] Life sciences
- [ ] Behavioural & social sciences
- [ ] Ecological, evolutionary & environmental sciences

For a reference copy of the document with all sections, see nature.com/documents/nr-reporting-summary-flat.pdf

Life sciences study design

All studies must disclose on these points even when the disclosure is negative.

Sample size	STORM image datasets were originally described in Otterstrom et al., Nucleic Acid Res 2019 (TSA Treatment) and Heo et al.,Nat. Biomed. Eng 2023 in which no sample size calculation has been performed before sample collection.
Data exclusions	Data corresponding to two nuclei of tendinosis, one nucleus of glass, and one nucleus for the control case of Y27 treatment have been excluded from the analysis. These data points were removed because they significantly deviate, based on a three-standard-deviation limit, from their respective datasets.
Replication	All experimental findings, including chromatin analysis and material characterization, were reliably reproduced.
Randomization	The cells and materials were randomly assigned to the treatment groups or conditions.
Blinding	The investigators were not blinded. All analyses were quantitative in nature, based on either established techniques or on techniques described in the Supplementary Information. There were no subjective or qualitative analyses where decision-making by the researchers would have been required or could have impacted the findings.

Reporting for specific materials, systems and methods

We require information from authors about some types of materials, experimental systems and methods used in many studies. Here, indicate whether each material, system or method listed is relevant to your study. If you are not sure if a list item applies to your research, read the appropriate section before selecting a response.

Materials & experimental systems

n/a	Involved in the study
☐	☒ Antibodies
☒	☐ Eukaryotic cell lines
☒	☐ Palaeontology and archaeology
☒	☐ Animals and other organisms
☒	☐ Clinical data
☒	☐ Dual use research of concern
☒	☐ Plants

Methods

n/a	Involved in the study
☒	☐ ChIP-seq
☒	☐ Flow cytometry
☒	☐ MRI-based neuroimaging

Antibodies

Antibodies used: Mouse anti-HDAC3 (1:50, Cell Signaling Technology, 3949), Anti-mouse Alexa Fluor 647(1:50, Abcam, 150107), DAPI (1 µg/ml, Thermo Fisher Scientific, D1306), Alexa Fluor 568 Phalloidin (15 nM, Thermo Fisher Scientific, A12380), Y27632 (10 µM, Abcam, 120129).

Validation: All antibodies were validated as described on the manufacturer's website or in our previously published studies.

Plants

Seed stocks: NA

Novel plant genotypes: NA

Authentication: NA
